# “Two hits - one stone”; increased efficacy of cisplatin-based therapies by targeting PCNA’s role in both DNA repair and cellular signaling

**DOI:** 10.18632/oncotarget.25963

**Published:** 2018-08-21

**Authors:** Caroline Krogh Søgaard, Augun Blindheim, Lisa M. Røst, Voin Petrović, Anala Nepal, Siri Bachke, Nina-Beate Liabakk, Odrun A. Gederaas, Trond Viset, Carl-Jørgen Arum, Per Bruheim, Marit Otterlei

**Affiliations:** ^1^ Department of Clinical and Molecular Medicine, Norwegian University of Science and Technology (NTNU), Trondheim, Norway; ^2^ Department of Urology and Surgery, St. Olavs Hospital, Trondheim University Hospital, Trondheim, Norway; ^3^ Clinic of Surgery, St. Olavs Hospital, Trondheim University Hospital, Trondheim, Norway; ^4^ Department of Biotechnology and Food Science, NTNU, Trondheim, Norway; ^5^ Department of Pathology, St. Olavs Hospital, Trondheim University Hospital, Trondheim, Norway; ^6^ APIM Therapeutics A/S, Trondheim, Norway

**Keywords:** muscle-invasive bladder cancer, APIM-peptide, cellular signaling, DNA repair, cisplatin resistance

## Abstract

Low response rate and rapid development of resistance against commonly used chemotherapeutic regimes demand new multi-targeting anti-cancer strategies. In this study, we target the stress-related roles of the scaffold protein PCNA with a cell-penetrating peptide containing the PCNA-interacting motif APIM. The APIM-peptide increased the efficacy of cisplatin-based therapies in a muscle-invasive bladder cancer (MIBC) solid tumor model in rat and in bladder cancer (BC) cell lines. By combining multiple omics-levels, from gene expression to proteome/kinome and metabolome, we revealed a unique downregulation of the EGFR/ERBB2 and PI3K/Akt/mTOR pathways in the APIM-peptide-cisplatin combination treated cells. Additionally, the combination treatment reduced the expression of anti-apoptotic proteins and proteins involved in development of resistance to cisplatin. Concurrently, we observed increased levels of DNA breaks in combination treated cells, suggesting that the APIM-peptide impaired PCNA - DNA repair protein interactions and reduced the efficacy of repair. This was also seen in cisplatin-resistant cells, which notably was re-sensitized to cisplatin by the APIM-peptide. Our data indicate that the increased efficacy of cisplatin treatment is mediated both via downregulation of known oncogenic signaling pathways and inhibition of DNA repair/translesion synthesis (TLS), thus the APIM-peptide hits both nuclear and cytosolic functions of PCNA. The novel multi-targeting strategy of the APIM-peptide could potentially improve the efficacy of chemotherapeutic regiments for treatment of MIBC, and likely other solid tumors.

## INTRODUCTION

BC is the ninth most common cancer worldwide, with an expected increase in incidence [1]. MIBC contributes to 30% of BC patients, and the 5-year survival rate after cystectomy is only 50% [2]. There have been few improvements in therapy since the advent of cisplatin. Immunotherapy via PD-1 inhibition is the only novel treatment recently accepted for MIBC, but the improvement in survival is so far modest [3]. Recent advances in genomic research have identified several therapeutic targets, however, their efficacy in therapy remains to be tested [4].

The gold standard in MIBC therapy is neoadjuvant cisplatin-containing treatment and cystectomy. Cisplatin-based chemotherapy is also the first line treatment for patients with metastatic disease, where gemcitabine/cisplatin (GC) and methotrexate/vinblastine/adriamycin/cisplatin (MVAC) are the main chemotherapeutic alternatives [2]. Formation of DNA interstrand crosslinks are responsible for the major cytotoxicity of cisplatin, but increased DNA repair, overexpression of ERBB2 and activation of the PI3K/Akt pathway often contributes to development of cisplatin resistance [5]. Cisplatin may offer longer survival, nonetheless, long term survival is uncommon in metastatic disease [6]. Cisplatin sensitization via strategies that can reduce cisplatin resistance can potentially improve metastatic as well as non-metastatic MIBC therapy.

PCNA acts as scaffold protein in several essential processes such as DNA replication, DNA repair and epigenetics [7, 8]. More recently, cytosolic scaffold roles of PCNA in apoptosis, glycolysis and signaling have been demonstrated [8–11]. The essential roles of PCNA during cellular stress and replication makes it a potential drug target, and a few PCNA-targeting drugs are under pre-clinical development [12].

The two known, and highly conserved, PCNA-interacting motifs, the PCNA-interacting peptide (PIP)-box and AlkB homologue 2 PCNA-interacting motif (APIM), are present in more than 600 proteins, and share the same binding site on PCNA [13–16]. Peptides and/or small molecules that bind with high affinity to this binding site will inhibit the majority of PCNA-protein interactions, and thereby inhibit essential cellular functions. Thus, such drugs will be cytotoxic to all cells. Accordingly, overexpression of a high affinity (canonical) PIP-box peptide is cytotoxic. On the other hand, overexpression of an APIM-peptide is well tolerated in the same cells in the absence of exogenous stress, but it strongly reduces cell growth and induces apoptosis in cells stressed with DNA damaging agents [10, 14, 17]. This is in line with the presence of APIM in many proteins involved in cellular stress responses, including the nucleotide excision repair (NER) protein XPA, the TLS polymerase POL ζ and proteins such as RAD51B, Topo IIa, TFII-I, ZRANB3 and FBH1, all which are important during replication stress and involved in repair of cisplatin-induced DNA lesions [14, 18–22]. Furthermore, the APIM-peptide is shown to enhance the efficacy of various chemotherapeutic drugs in multiple cancer cells both *in vitro* and *in vivo*, i.e. i) in a multiple myeloma xenograft model and an endogenous orthotopic prostate cancer model after intraperitoneal administration in combination with melphalan and docetaxel [10, 23], ii) in both syngeneic and endogenous orthotopic non-MIBC models in rats after intravesical administrations in combination with mitomycin C [24]. Several lines of evidence indicate that the chemo-sensitizing effect of the APIM-peptide is caused by the direct binding of the APIM-peptide to PCNA and that APIM-PCNA interactions are stronger under cellular stress and at least partly mediated by posttranslational modifications on PCNA [8, 10, 14, 18, 19, 22, 25].

Here we show that the APIM-peptide enhances the anti-cancer efficacy of cisplatin in a syngeneic orthotopic MIBC model in rats and increases the efficacy of GC and MVAC in a panel of human BC cell lines. The APIM-peptide-cisplatin combination reduces the expression of multiple proteins and oncogenic pathways, often upregulated in BC as well as in other solid tumors. We detect increased levels of DNA strand breaks after APIM-peptide-cisplatin treatment, suggesting that the APIM-peptide inhibits repair of cisplatin-induced lesions. Notably, the APIM-peptide re-sensitizes cisplatin-resistant BC cells and elevates the levels of DNA strand breaks in these cells to the same level as in cisplatin-sensitive cells.

## RESULTS

### APIM-peptide increased the anti-cancer efficacy of cisplatin *in vivo*

**Figure 1:**
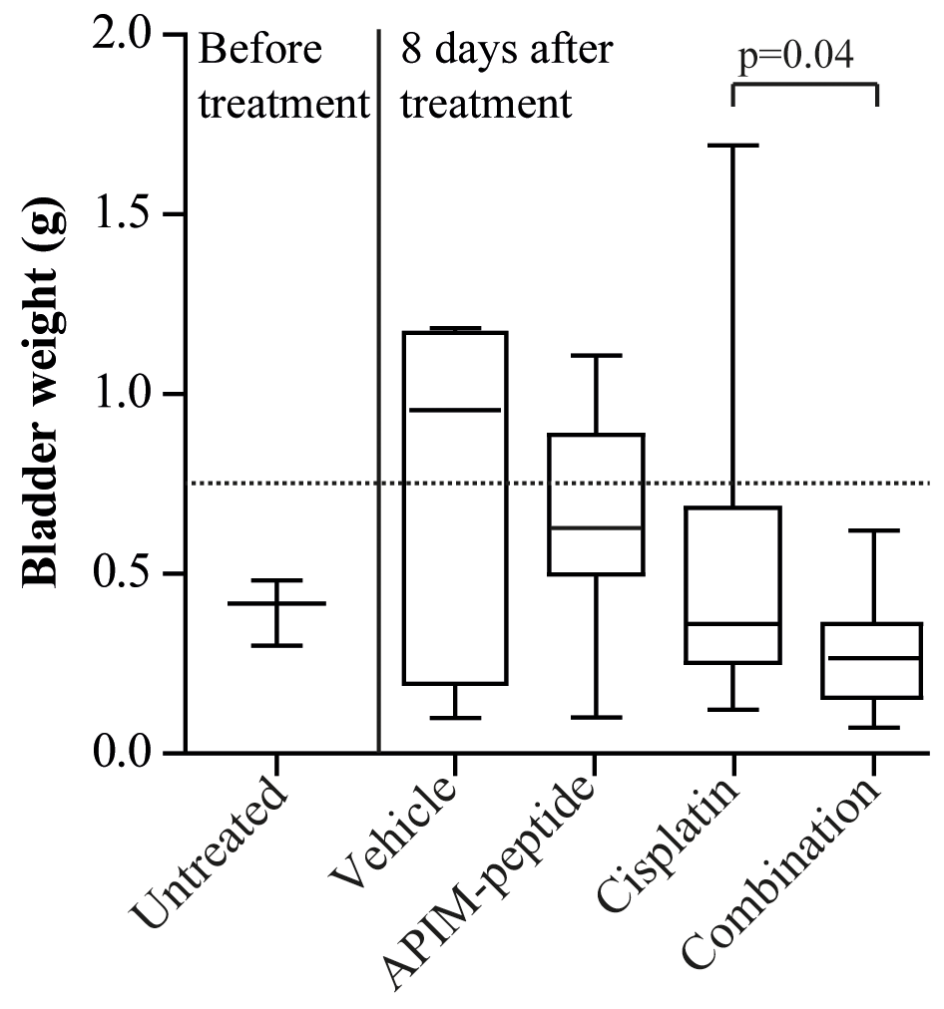
Combination of APIM-peptide and cisplatin therapy inhibits tumor growth in an orthotopic MIBC solid tumor model Box-and-whisker plot of rat bladder weights harvested before treatment (n=3) or eight days after intravenous treatment with vehicle (NaCl, 0.9%, n=7), APIM-peptide (8.5 and 12.5 mg APIM-peptide peptide/kg, n=7) and cisplatin (2 mg/kg, n=16) alone or in combination (n=19). Data from three biological replicas is included in the figure. P-values were calculated using unpaired, two-tailed student *t*-test. The line in the box is the median, the box extends from the lower to the upper quartile, and the whiskers represent the lowest and highest bladder weight in each group.

The anti-cancer effect of the APIM-peptide in combination with cisplatin was first examined in a MIBC model in rat. Inoculated cells were left to grow for three weeks before three rats were terminated to establish that the instilled cells had progressed to MIBC (untreated, Figure 1). Histopathological evaluation confirmed that two of these bladders had muscle invasive high grade (T2G3) tumors at this time point, while the last was classified as non-muscle invasive high grade (T1G3) (Table 1A). We therefore treated the remaining rats at this time point and evaluated treatment efficacy one week later.

**Table 1:** Histopathological classification of bladders before and after therapy

A		
Bladder weight (g)	Histological classification	Clinical classification
**0.30**	T2G3	MIBC
**0.48**	T2G3	MIBC
**0.42**	T1G3	NMIBC

B			
Cisplatin	APIM-peptide-cisplatin		
Bladder weight (g)	Histological classification	Bladder weight (g)	Histological classification
0.12	Normal	0.07	TaG2
0.22	T1G3	0.08	Normal
0.23	T1G2	0.09	Normal
0.23	T1G2	0.12	TaG2
0.27	T2G3	0.16	TaG1
0.29	T1G2	0.21	T2G3
0.33	T2G3	0.23	TaG1
0.36	T2G3	0.24	T2G3
0.36	T2G3	0.27	T1G3
0.39	T2G3	0.27	T1G3
0.43	T3G3	0.27	T1G2
0.57	T3G3	0.31	TaG2
0.61	TaG1	0.32	T3G3
0.76	T3G3	0.36	T2G3
0.86	T3G3	0.36	T2G3
1.69	T3G3	0.41	T2G3
**Average: 0.48**		0.47	T2G3
		0.48	T3G3
		0.62	T3G3
		**Average: 0.28**	

Histopathological evaluation of rat bladders inoculated with AY-27 cells for three weeks at **(A)** treatment start and **(B)** 8 days after treatment with cisplatin (2 mg/kg) as single agent or in combination with APIM-peptide (8.5 mg/kg or 12.5 mg/kg APIM-peptide/kg). Normal bladder weight is approximately 0.1 g, while tumor-containing bladders are heavier corresponding to size and grade of tumor [24].

Effect of the treatment was defined as bladder weight lower than the average bladder weight of the vehicle group (broken line in Figure 1). Effect of treatment was found in 100% of the combination group, compared to 81% of the cisplatin group and 43% of the APIM-peptide group (29% in vehicle group). Importantly, the combination group had a significantly lower tumor weight (p=0.04) and a more uniform response to treatment than the cisplatin group (Table 1B and Figure 1). Of notice, no acute toxicity was observed in rats treated with the APIM-peptide. Histopathological evaluation of the bladders confirmed fewer invasive tumors (T2/3G3) in the combination group (47%) than in the cisplatin group (63%) (Table 1B). Because the initial tumor volume in individual rats prior to treatments is unknown in this model, it was difficult to establish whether bladders classified as histopathological “normal” were cured, or if they were non-takes (one in cisplatin and two in combination group, see Table 1B). However, the bladder weights were significantly lower in the combination group than in the cisplatin group even if the cured/potential non-takes were excluded (p=0.05). Our results suggest that the APIM-peptide can potentiate the anti-cancer efficacy of cisplatin.

To explore the biodistribution of APIM-peptide after *i.v*. infusion, we harvested tissue from thigh, heart, kidney, brain, liver and bladder immediately after infusion of fluorescently tagged APIM-peptides. Positive fluorescence was detected by confocal imaging in frozen sections from all organs evaluated, including the bladder, supporting that the increased anti-cancer activity of cisplatin on bladder tumors was due to the presence of the APIM-peptide (Supplementary Figure 1).

### Efficacy of cisplatin-containing treatments were enhanced by the APIM-peptide *in vitro*

**Figure 2:**
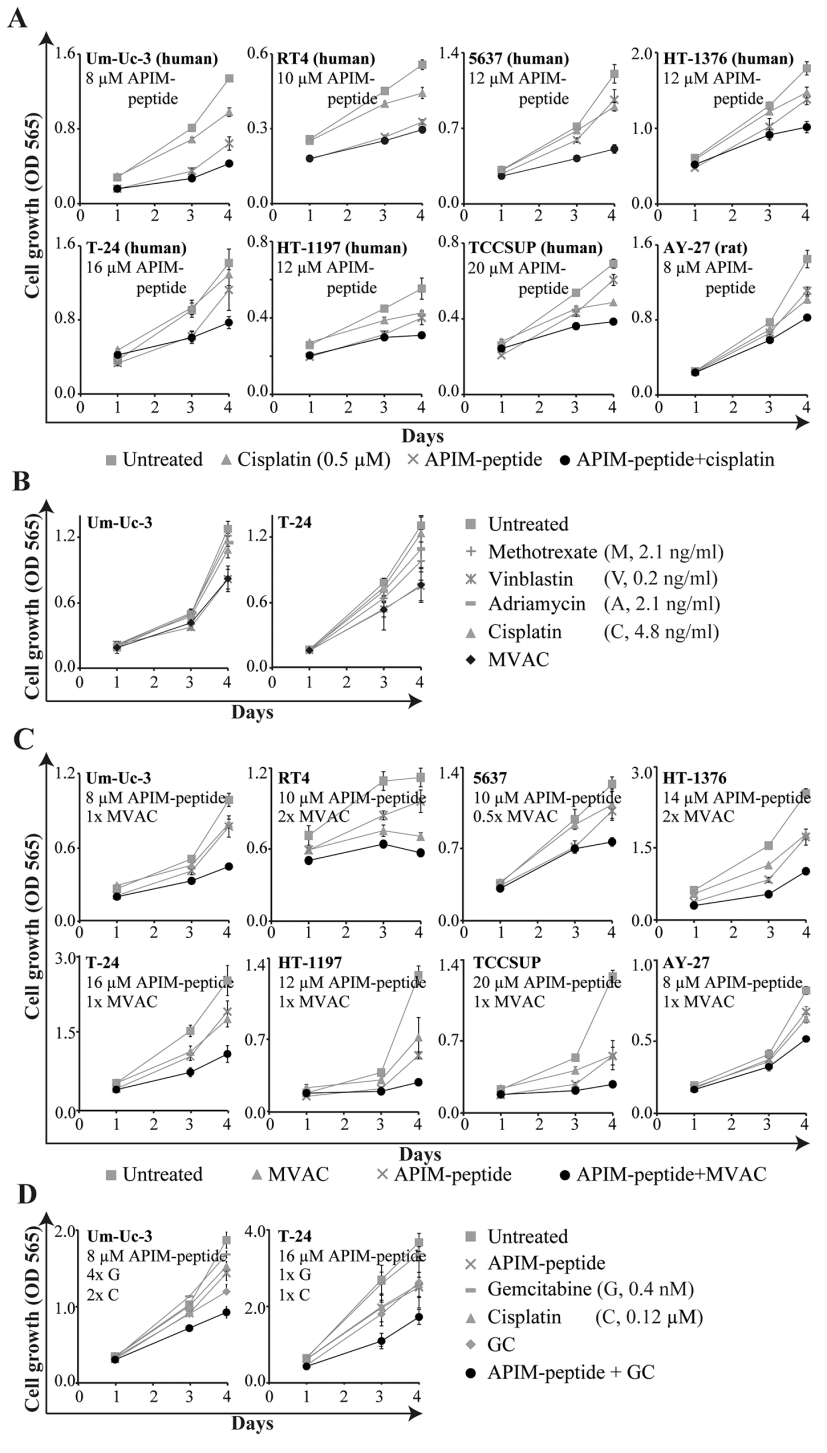
APIM-peptide in combination with clinically relevant cisplatin-containing combinations inhibits cell growth *in vitro* Cell growth (MTT assay) of BC cell lines after continuous exposure to the APIM-peptide and chemotherapeutic agents as single agents or in combination (added on day 0). Each graph is from one representative replica of at least three biological replicas. Data is displayed as average ± SD (4-6 technical replicas). **(A)** APIM-peptide and cisplatin as single agents and in combination. **(B)** Methotrexate, vinblastine, adriamycin and cisplatin as single agents and in combination (MVAC). The sensitivity to vinblastine as a single agent are similar as to MVAC. **(C)** APIM-peptide and MVAC (0.5-2x of the doses in (B)) alone and in combination. **(D)** APIM-peptide, gemcitabine and cisplatin as single agents and in combinations (GC).

Next, we examined if the APIM-peptide could increase the sensitivity of several cisplatin-containing treatments using a panel of human BC cells. Previously, we found that the sensitivity towards the APIM-peptide as a single agent varied in these cell lines, but that this was not linked to their PCNA levels [24]. However, their sensitivities towards cisplatin were similar and, importantly, the efficacies of cisplatin, MVAC and GC were enhanced by the APIM-peptide in all cell lines (Figure 2). Our results suggest that the APIM-peptide increases the efficacies of several chemotherapeutics used for MIBC therapy.

### APIM-peptide-cisplatin treatment increased the number of differentially expressed (DE) genes

**Figure 3:**
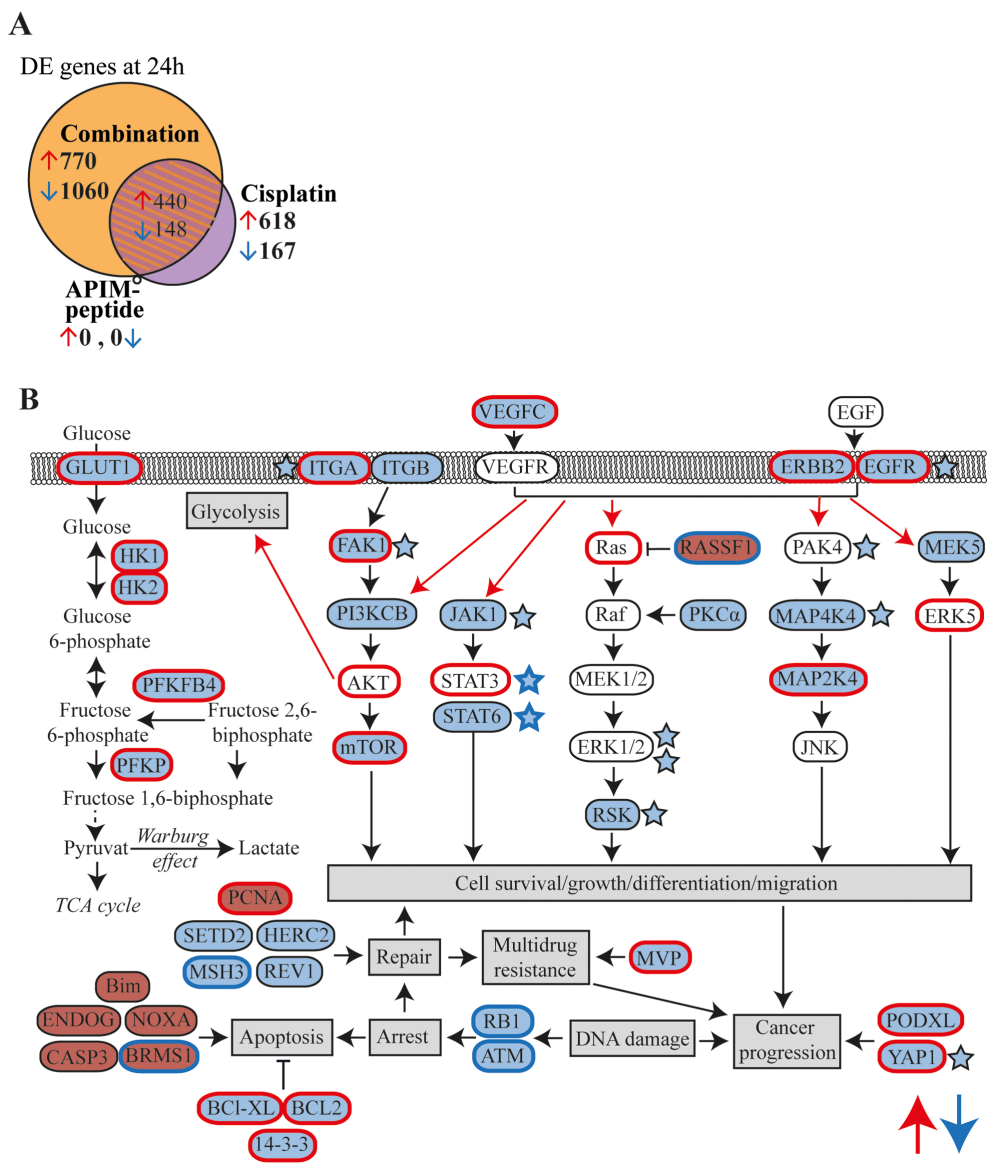
APIM-peptide in combination with cisplatin downregulates expression of frequently overexpressed genes in MIBC Microarray analysis on Um-Uc-3 and T-24 cells treated for 24h with APIM-peptide (8 and 16 μM, respectively) and cisplatin (10 μM) alone or in combination (n=6). **(A)** Venn diagram illustrating number of differentially expressed (DE) genes in each treatment group (relative to untreated control, FC>1.25). Orange area marks the part unique for the combination group. **(B)** Schematic overview highlighting the most interesting upregulated (red background) and downregulated (blue background) DE genes detected only in the combination group with relevance to MIBC. Red edge = often overexpressed in MIBC, blue edge = often inactivated in MIBC, red arrows = often upregulated pathways in MIBC [4, 26-30, 32, 33, 37, 49-54]. Stars denote downregulated proteins detected by the MIB-assay; i) only in the combination group (blue stars with blue edges) or ii) more downregulated in the combination group than the cisplatin group (blue stars black edges).

We selected the Um-Uc-3 and T-24 cell lines for gene expression analysis because they represent one APIM-peptide single agent sensitive (Um-Uc-3) and one insensitive (T-24) cell line. Still, APIM-peptide treatment increased the efficacy of cisplatin in both cell lines. We only included DE genes similarly changed in all three biological replicas of both cell lines. The APIM-peptide as a single agent did not have any similar effects on gene expression in the two cell lines (Figure 3A). Cisplatin as a single agent altered gene expression of multiple genes similarly in the two cell lines, and 75% of these DE genes overlapped with those in the APIM-peptide-cisplatin treated group. However, the combination treatment resulted in more than 1200 additional DE genes not affected by cisplatin treatment alone (combination minus shaded area, Figure 3A). The majority of these genes were downregulated (73%). Similar trends were seen after 4 hours of treatment, but the number of DE genes were strongly increased from 4 to 24 hours (Supplementary Figure 2).

### Combination treatment downregulated genes frequently overexpressed in cancer

The DE genes identified only in the combination group (orange area in Figure 3A, gene lists in Supplementary Table 1) were subjected to gene enrichment analysis. Alterations in several pathways including cell cycle, DNA damage, EGFR/VEGF signaling, transcription and apoptosis were identified (Table 2). A simplified schematic overview highlighting DE genes after combination treatment in relation to the most relevant pathways for MIBC are shown in Figure 3B. Expression of *VEGFC, EGFR, ERBB2* and several genes encoding proteins in downstream MAPK and PI3K/Akt signaling pathways were downregulated. Interestingly, these are commonly overexpressed in MIBC, as well as other solid cancers [26, 27]. Furthermore, downregulation of several genes encoding proteins involved in the DNA damage response, e.g. RB1, ATM, HERC2 (NER), REV1 (TLS), MSH3 (mismatch repair) and SETD2 (homologues recombination) were detected. Downregulation of glycolysis was indicated by the reduced expression of *GLUT1, HK1/2* and other glycolytic enzymes often overexpressed in BC [28]. Moreover, pro-apoptotic factors such as Bim and caspase 3 were upregulated, while anti-apoptotic factors such as BCL2 and BCL-XL, commonly overexpressed in BC [26], were downregulated. Our results demonstrate that combination treatment alters key genes in MIBC that are supportive of the inhibited BC growth observed both *in vivo* (Figure 1) and *in vitro* (Figure 2).

**Table 2:** Gene enrichment indicates altered cell cycle regulation and signaling by the APIM-peptide-cisplatin combination at 24h

GeneGo pathway map		Genes in pathway	False discovery Rate (FDR)
**Upregulated:**			
**Cell cycle**			
1.	Role of APC in cell cycle regulation	6/32	5E-5
3.	Transition and termination of DNA replication	4/28	5E-3
7.	Role of SCF complex in cell cycle regulation	3/29	5E-2
10.	Start of DNA replication in early S phase	3/32	5E-2
**Transcription**			
2.	Assembly of RNA Polymerase II preinitiation complex on TATA-less promoters	4/18	1.4E-3
5.	Huntington-depended transcription deregulation in Huntington's Disease	3/24	4E-2
**Apoptosis and survival**			
4.	p53-dependent apoptosis	4/29	5E-3
**DNA damage**			
6.	ATM / ATR regulation of G2 / M checkpoint	3/26	5E-2
9.	ATM/ATR regulation of G1/S checkpoint	3/32	5E-2
**Metabolism**			
8.	CTP/UTP metabolism	5/108	5E-2
**Downregulated:**			
**Cytoskeleton remodeling**			
1.	TGF, WNT and cytoskeletal remodeling	16/111	4E-4
5.	Cytoskeleton remodeling	14/102	9E-4
**Signaling**			
2.	HBV signaling via protein kinases leading to HCC	9/36	4E-4
9.	Regulation of p38 and JNK signaling mediated by G-proteins	8/39	3E-3
**Development**			
3.	Growth factors in regulation of oligodendrocyte precursor cell survival	9/37	4E-4
4.	PIP3 signaling in cardiac myocytes	10/47	4E-4
14.	EGFR signaling via small GTPases	7/33	3E-3
16.	VEGF signaling via VEGFR2 - generic cascades	11/84	3E-3
17.	Cytokine-mediated regulation of megakaryopoiesis	9/57	3E-3
**Transport**			
6.	Clathrin-coated vesicle cycle	11/71	2E-3
**Cell adhesion**			
7.	Chemokines and adhesion	13/100	2E-3
19.	Histamine H1 receptor signaling in the interruption of cell barrier integrity	8/45	3E-3
20.	Ephrin signaling	8/45	3E-3
**Neurophysiological process**			
8.	Main pathways of Schwann cells transformation in neurofibromatosis type 1	10/62	2E-3
18.	Receptor-mediated axon growth repulsion	8/45	3E-3
**Muscle-contraction**			
10.	S1P2 receptor-mediated smooth muscle contraction	7/30	3E-3
**Translation**			
11.	Translation regulation by Alpha-1 adrenergic receptors	9/53	3E-3
**Apoptosis and survival**			3E-3
12.	BAD phosphorylation	8/42	3E-3
13.	Anti-apoptotic action of Gastrin	8/43	3E-3
**Cell cycle**			
15.	ESR1 regulation of G1/S transition	7/33	3E-3

List of all significant upregulated and top 20 significant downregulated GeneGo pathway maps. The gene enrichment analysis were done on the differentially expressed genes (fold change > 1.25 relative to control, and found in all six biological replica of Um-Uc-3 and T-24 cells) unique for the APIM-peptide-cisplatin combination group, and not detected in cisplatin or APIM-peptide single agent groups (lists of genes in Supplementary Table 1). The GeneGo pathway maps are grouped by their main category.

### APIM-peptide enhanced cisplatin-induced changes in cellular signaling

**Figure 4:**
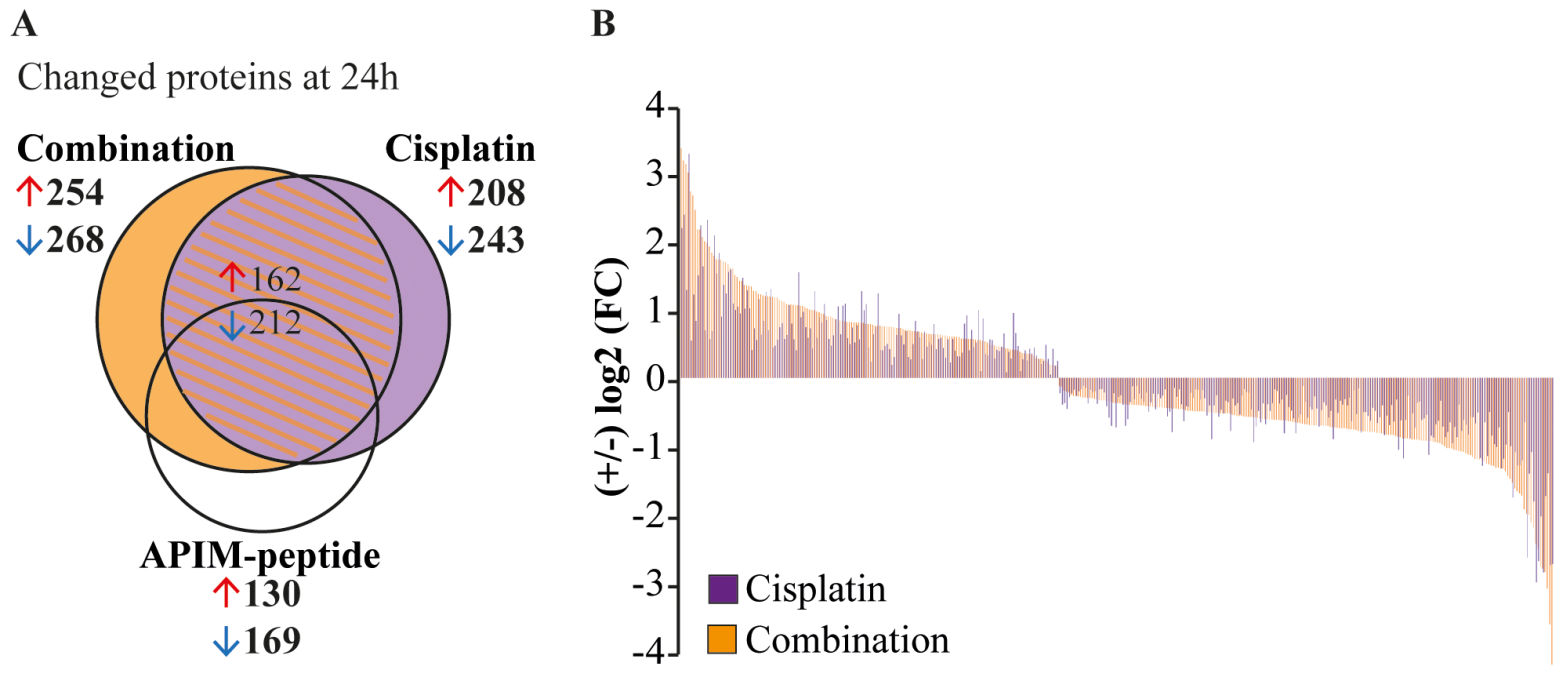
APIM-peptide enhances protein changes induced by cisplatin Significantly changed proteins measured using the MIB-assay (Wilcoxon Sign Rank test, p<0.25) in Um-Uc-3 and T-24 cells treated for 24h with APIM-peptide (8 and 16 μM, respectively) and cisplatin (10 μM) (relative to untreated control). **(A)** Venn diagram illustrating the number of changed proteins in each treatment group. **(B)** Log2 fold change (FC) of proteins detected in both cisplatin and the combination group. Each protein presented by one bar, only proteins with >5% difference in relative values of combination (orange bars) vs cisplatin (purple bars) are shown.

To confirm the alterations in cellular signaling indicated by gene expression analysis on protein level, we enriched the cell extracts from Um-Uc-3 and T-24 for kinases and other dNTP/NTP interacting proteins prior to mass spectrometry (MS) analysis using the multiplexed inhibitor bead (MIB)-assay. We detected significant changes in 522 proteins after APIM-peptide-cisplatin treatment compared to untreated control (Figure 4A). This included 4 phosphatases, 15 ubiquitin ligases and other proteasome/chaperone proteins as well as 32 signaling kinases. Of these proteins, 148 were unique for the combination group (orange area in Figure 4A, protein lists in Supplementary Table 2). Many of the same proteins were pulled down in all treatment groups, however, 67% of the proteins pulled down in both cisplatin and combination groups (shaded area Figure 4A) were more increased/reduced by the combination treatment (Figure 4B). Reduced pull-down of multiple proteins in the combination group supported downregulation of the EGFR/ERBB2, MAPK and PI3K/Akt pathways as suggested by the gene expression analysis (stars in Figure 3B).

### APIM-peptide-cisplatin combination increased glucose and glutamine consumption and affected central carbon metabolism

**Figure 5:**
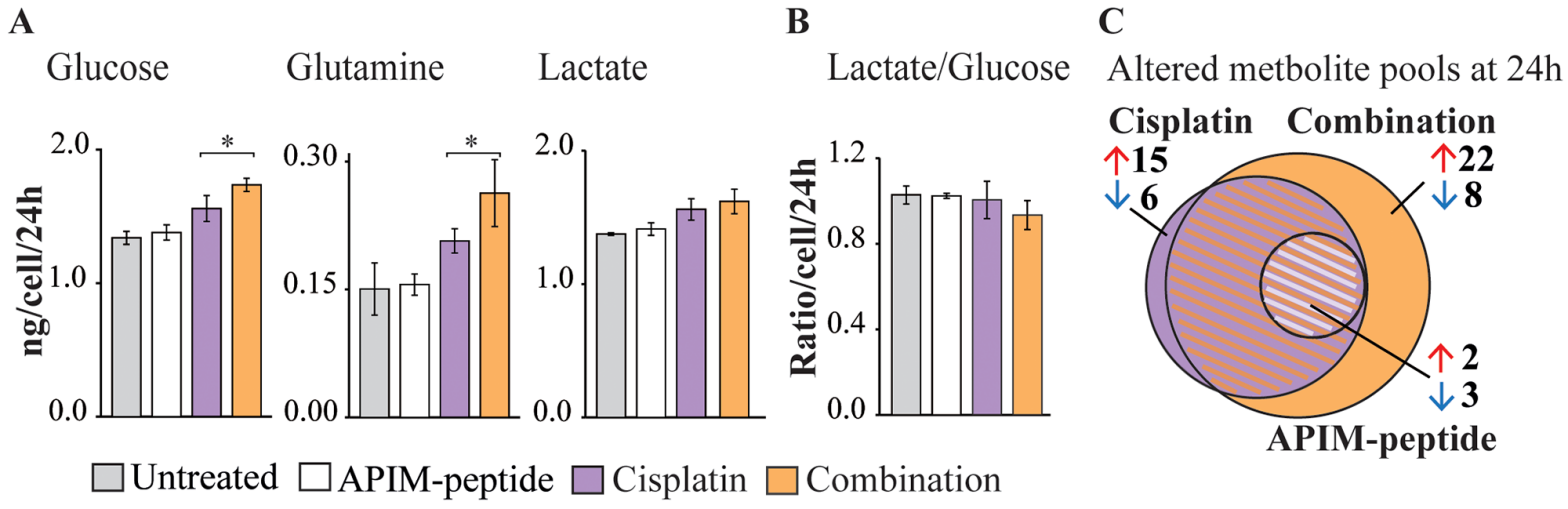
APIM-peptide-cisplatin combination increases energy source consumption and affects central carbon metabolism Consumption/excretion of extracellular metabolites and targeted metabolic profiling of T-24 cells treated for 24 hours with APIM-peptide (16 μM), cisplatin (10 μM) and the combination (n=4). **(A)** Glucose and glutamine consumption and lactate excretion per live cell per 24 hours in each treatment group ± SD. Significant (^*^p<0.05) and non-significant (ANOVA and post hoc Tukey’s range test) differences between cisplatin and APIM-peptide-cisplatin treated cells are indicated. Combination and cisplatin treated cells were significantly different from untreated control in all, while APIM-peptide single agent treatment was not (not marked in figure). **(B)** Lactate/glucose ratio per live cell per 24 hours in each treatment group ± SD. **(C)** Venn diagram illustrating the number of significantly (ANOVA and post hoc Tukey’s range test, p<0.05) changed intracellular central carbon metabolite pools relative to control) in each treatment group.

Gene expression analysis indicated that the APIM-peptide-cisplatin combination downregulates genes encoding glycolytic enzymes. To investigate whether these changes were reflected in the metabolome we next measured glucose and glutamine consumption, lactate production and applied targeted metabolic profiling of central carbon metabolism. We detected low residual glucose in Um-Uc-3 cell cultures, and even though addition of glucose in control experiments did not affect cell growth or sensitivity to treatment (Supplementary Figure 3), it could cause altered carbon metabolism. Therefore, emphasis was placed on metabolic responses in T-24 cells, although most trends were reproduced in Um-Uc-3 cells (Supplementary Figure 4B, and bolded in 4C). APIM-peptide-cisplatin treatment significantly increased glucose and glutamine consumption compared to cisplatin as a single agent. Lactate excretion was increased in both cisplatin and combination treated cells, yet the lactate/glucose ratio was decreased in combination treated cells only (Figure 5A–5B). The reduced ratio, although not significant, suggests that the APIM-peptide reduces the Warburg effect in cisplatin treated cells.

The altered glucose and glutamine consumption of cisplatin and APIM-peptide-cisplatin treated cells was reflected intracellularly by several significantly changed metabolite pool sizes (Supplementary Figure 4). Common to both treatments was increased levels of essential amino acids and deoxynucleosides, likely attributed to growth arrest and inhibition of replication. The combination treatment evoked larger changes in more metabolite pools than cisplatin as a single agent (Figure 5C, “+” in Supplementary Figure 4C). The most prominent changes were a buildup of metabolites after the rate-limiting conversion of fructose-6 phosphate to fructose 1,6-bisphosphate in glycolysis, a reduction of the 6-phospoglyconate pool in the entry to pentose phosphate pathway (PPP) and a reduction in the α-ketoglutarate pool of tricarboxylic acid (TCA) cycle (Supplementary Figure 4C). Altogether, the upregulated glucose and glutamine consumption, reduced lactate/glucose ratio and altered metabolite pool sizes at important metabolic branch points shows that BC cells undergo considerable changes in central carbon metabolism as a response to the APIM-peptide-cisplatin combination therapy. However, an exact explanation for the anti-cancer activity observed requires further studies.

### APIM-peptide re-sensitized cisplatin resistant cells

**Figure 6:**
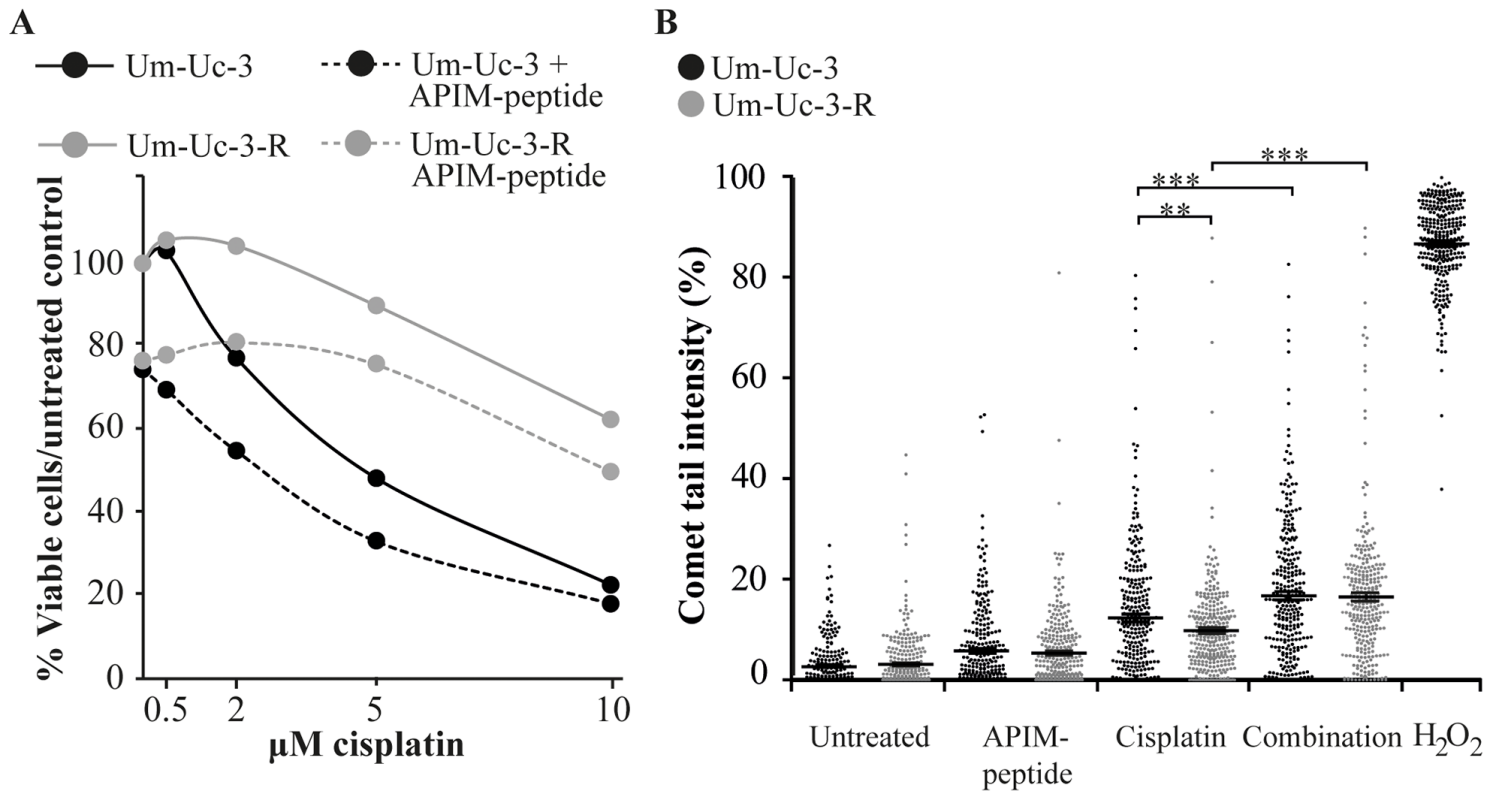
APIM-peptide re-sensitizes cisplatin-resistant cells Original Um-Uc-3 and cisplatin-resistant Um-Uc-3-R cells treated with the APIM-peptide (8 μM) and cisplatin (**A:** 0.5-10 μM, **B:** 10 μM). **(A)** Dose-response of treated cells relative to untreated cells measured by the MTT assay after 48 hours of continuous exposure to treatments. Data presented is one representative experiment out of at least three biological replicas. **(B)** Percentage tail intensity of comets from alkaline comet assay analysis after 24h exposure to treatments. H_2_O_2_ (100 mM) was used as positive control. Data is merged from three biological replica in which 100 comets were randomly selected from each experiment (n=300), and presented as scatter plot with mean ± SEM. ^**^p<0.01, ^***^p<0.0001 (student-*t* test, two tailed). All treatments were significantly (^***^) different from untreated control, and all single treatments were significantly (^***^) different from combination treatments (not marked in Figure).

Development of resistance is a major problem in cancer therapy and the mechanisms are multifactorial, including enhanced DNA repair, impaired signaling and reduced intracellular cisplatin accumulation [5]. Gene expression analysis indicated that the APIM-peptide-cisplatin treatment downregulated expression of *PODXL, YAP1* and *MVP* (Figure 3B); genes that are commonly overexpressed in MIBC and associated with multidrug resistance [4, 29, 30]. We therefore developed a cisplatin resistant Um-Uc-3 cell line (Um-Uc-3-R) and investigated the effect of the APIM-peptide on cisplatin sensitivity in this cell line. Um-Uc-3-R, cells were more resistant to cisplatin compared to original Um-Uc-3 cells at all doses tested and importantly, the APIM-peptide increased the sensitivity of both Um-Uc-3 and Um-Uc-3-R cells (Figure 6A, viability after 48 hours exposure). For instance, the viability of Um-Uc-3-R cells was not reduced by 2 μM cisplatin, while the viability of Um-Uc-3 cells was reduced with 20% at this time point. However, when combined with the APIM-peptide, the Um-Uc-3-R cells were re-sensitized to this dose of cisplatin (Figure 6A).

To explore the molecular mechanism behind this sensitizing effect, we examined if the APIM-peptide increased the levels of DNA lesions by impairing DNA repair in cisplatin treated cells. All treatments significantly increased the level of DNA damage relative to untreated control in both original Um-Uc-3 and cisplatin-resistant Um-Uc-3-R cells. In accordance with lower cisplatin sensitivity, Um-Uc-3-R cells had lower levels of DNA damage than Um-Uc-3 cells treated with the same dose of cisplatin after 24 hours (Figure 6B). However, the combination of cisplatin and APIM-peptide increased the amount of DNA damage in both these two cell lines and leveled out the differences between them. This indicates that at least part of the APIM-peptide re-sensitizing effect is mediated via inhibition of DNA repair. Multiple APIM-containing proteins, such as XPA and polymerase ζ, are directly involved in bypass or repair of cisplatin-induced DNA lesions and could be inhibited by APIM-peptide treatment, in support for this finding. Furthermore, expression of HERC2 and REV1, also important for NER and TLS, were downregulated in combination treated cells (Figure 3B) and could also contribute to the increased level of DNA lesions observed.

Next we analyzed Um-Uc-3 and Um-Uc-3-R cells for cell cycle effects and fraction of apoptotic cells upon treatment with cisplatin and the cisplatin-APIM-peptide combination. Both cell lines were arrested to the same extent in S-phase and no significant changes could be detected between the cell lines after 24 hours (Supplementary Figure 5A). The APIM-peptide increased the fraction of apoptotic cells after cisplatin treatment in Um-Uc-3 while apoptosis was not affected by any of the treatments in Um-Uc-3-R cells (Supplementary Figure 5B). Thus, there is no direct link between increased level of DNA damage induced by the combination treatment and an increase in apoptosis in the Um-Uc-3-R cells at 24 hours. Both the cisplatin alone and the combination treatment did cause a small reduction in viability for both cell lines at this time point, and in accordance with the apoptosis data it was greater for Um-Uc-3 than for the Um-Uc-3-R cells (Supplementary Figure 5C). The reduction in viability and difference between the cell lines was further enhanced after 48 hours (Figure 6A, 10 μM cisplatin), suggesting a delayed and/or reduced DDR response in the Um-Uc-3-R cells.

## DISCUSSION

Our results demonstrate that the PCNA-interacting APIM-peptide increases the anti-cancer efficacy of cisplatin *in vivo* by reducing tumor load and down staging BC, and thus has the potential to improve MIBC therapy. This is supported by previous work showing that the APIM-peptide is able to increase the efficacy of mitomycin C on non-MIBC [24]. Furthermore, this study reveals DE of apoptotic genes, changes in glycolytic enzymes and metabolites, and alterations in several signaling pathways often involved in oncogenic transformation when cisplatin is combined with the APIM-peptide. The exact same changes were not identified on all omics levels, however, this was expected because the genome, proteome and metabolome are highly dynamic, and not necessarily in phase at a given time point. Further, we discuss only a subset of the altered genes, proteins and metabolites in our data sets because exploring the complete system-biological effects of treatment via integrating all omics levels requires dedicated computational tools. Time series and further computational analysis will be the focus of future work. Yet, we demonstrate that many predicted therapeutic targets in BC are affected by the APIM-peptide-cisplatin treatment on more than one omic level, and that the changes observed are in in accordance with the observed *in vivo* and *in vitro* anti-cancer effects.

In this study, we show that APIM-peptide-cisplatin treatment leads to changed expression of multiple proteins implicated in cancer cell growth and development of cisplatin resistance. Combination treated cells reduced the expression of genes encoding proteins in the DNA damage response, both in cellular signaling, NER and TLS, and had increased levels of DNA damage. In addition to the changes in gene expression, the APIM-peptide likely directly inhibits NER and TLS as APIM-containing protein in these pathways are dependent on interaction with PCNA for optimal function [18, 22]. EGFR, ERBB2 and members of the downstream PI3K/Akt and Ras pathways are potential therapeutic cancer targets, and they were all downregulated by the combination treatment. Mutations in these pathways are found in over 40% of BC tumors and inhibitors of these are suggested to restore cisplatin sensitivity [4, 31]. It is difficult to determine whether it is the direct inhibition of NER or TLS, or the effects on the EGFR/ERBB2 and downstream pathways or both that is responsible for the re-sensitization of cisplatin resistant cells observed after APIM-peptide-cisplatin treatment. Most likely the re-sensitizing effect is a combination of multiple factors.

The APIM-peptide-cisplatin combination also reduced the levels of *JAK, STAT* and *FAK1* expression. STAT3 and FAK1 activation are reported to be important in multiple cancer types, including BC [32, 33]. Although several inhibitors targeting EGFR, MAPK, FAK1 or PI3K/Akt pathways are undergoing clinical trials for MIBC therapy, no drug has yet been approved for BC treatment [32, 34–36]. RASSF1 is suggested to have tumor suppressor functions through inhibition of the Ras pathway. Reduced expression due to hypermethylation is frequently observed in BC (∼80%), and is associated with progression and shorter overall survival [37]. Interestingly, the APIM-peptide-cisplatin treatment increased RASSF1 expression. This effect could be mediated via inhibition of PCNA´s role in signaling, however, many proteins involved in regulation of DNA methylations e.g. the TET-proteins and DNA methyl transferases contain PCNA interacting motifs [14]. Therefore, increased RASSF1 could also be due to APIM-peptide mediated inhibition of DNA methylation.

It is not straightforward to predict the most prominent effects of the APIM-peptide in specific cancer cells because more than 300 proteins involved in multiple signaling and DNA damage pathways contain APIM. All of these potential PCNA interactions might be more or less impaired, although not identically in cells of different origin. The dependence on, and regulation of, different cellular pathways varies between cells of different tissue origins, as well as between normal and cancer cells. In any case, targeting PCNA with the APIM-peptide has the potential to affect, i.e. partly impair, but not completely inhibit, multiple pathways important in cellular stress responses simultaneously. Because cancer cells are more dysregulated and often lack normal check point regulation, this stress-confined treatment strategy is shown to have larger impact on cancer cells than normal cells across a range of cancer subtypes [8, 10]. This treatment strategy is less likely to be circumvented by development of resistance because it targets multiple pathways, and by itself targets TLS and therefore reduces mutagenicity [22].

## MATERIALS AND METHODS

### Cell lines

The syngeneic rat urothelial carcinoma cell line AY-27 used in the *in vivo* studies was kindly provided by Professor S. Selman, Department of Urology, Medical College of Ohio and grown as described [38]. A panel consisting of the human urothelial carcinoma cell lines TCCSUP, HT-1197, Um-Uc-3, HT-1376, RT4, T-24 and 5637 (ATCC No. TCP-1020) were used for the *in vitro* studies. All cells were grown as recommended and cultivated in a humidified atmosphere (5% CO_2_, 37°C). Additionally, a cisplatin resistant Um-Uc-3 cell line (Um-Uc-3-R) were established by continuously exposing the cells to increasing doses of cisplatin over one year (0.0625-1 μM cisplatin, added twice a week).

### Treatment agents

APIM-peptide (ATX-101, MD-RWLVK-W-KKKRK-I-RRRRRRRRRRR) (APIM Therapeutics, Bachem) [10], cisplatin (Hospira), methotrexate (Pfizer), vinblastine (Velbe), adriamycin (Pfizer), gemcitabine (Santa Cruz Biotechnology) and hydrogen peroxide (Sigma-Aldrich).

### Animals and ethics

The study was approved by the Norwegian National Animal Research Authority (Forsøksdyrutvalget, FDU) (FOTS applications 5502 and 6842) and in accordance with Norwegian and EU guidelines for care and use of laboratory animal.

Female CDF344 rats (Harlan Laboratories, Blackthorn) were kept in a standardized environment. Rats were anesthetized (subcutaneously) with a mixture (0.35-0.40 mL/100 g body weight (BW)) consisting of haloperidol (5 mg/mL, Janssen) (17% v/v), fentanyl (50 μg/mL, Actavis) (25% v/v) and midazolam (5 mg/mL, Actavis) (25% v/v) before orthotopic implantation. After implantation, the rats received NaCl (0.9%, 5-10 mL) and temgesic (0.3 mg/mL, 0.33 mL/200 g BW, RB Pharmaceuticals Ltd.) subcutaneously if needed, as judged by their condition. Intravenous (i.v.) treatment was performed under general anesthesia with isoflurane (4% induction, 1.5-2% maintenance). Anaesthetized rats were kept on a heat blanket to maintain body temperature. The rats were monitored for general health status and BW throughout the duration of the experiments.

### *In vivo* MIBC model

The *in vivo* studies were performed with an immunocompetent rat orthotopic BC model previously described with the instillation of 4x10^5^ AY-27 rat BC cells [38, 39]. The rats were kept for three weeks to establish muscle-invasive tumors before treatment [40]. The rats were randomly distributed into treatment groups; i) vehicle (NaCl, 0.9%), ii) APIM-peptide (8.5 or 12.5 mg net APIM-peptide/kg), iii) cisplatin (2 mg/kg) and iv) APIM-peptide-cisplatin combination. First, cisplatin was given intravenously with a syringe (0.4 mL over 2 min), and the APIM-peptide was given subsequently via i.v. infusions using a pump (Aleris Guardrails Rolle) to ensure accuracy (2.4 mL/h, 12.5 mg/kg BW/mL) (rats in vehicle and cisplatin group were given saline infusions). The rats were treated once and the bladders were harvested after eight days. The bladders were macroscopically evaluated, weighed and stored in buffered formaldehyde solution (4%) until processing for histopathological evaluation. Statistical significance between the cisplatin and APIM-peptide-cisplatin groups was calculated using student *t*-test (unpaired, two-tailed, p<0.05).

In total, 57 rats from three independent biological replicas were used in this study. Of these, 5 rats are not included in Figure 1: i) three rats died before treatment, ii) one NaCl-treated rat died due to large tumor, iii) one rat was terminated before treatment due to reduced health status. The APIM-peptide and cisplatin combination treated groups with 8.5 or 12.5 mg APIM-peptide/kg were combined as there were no difference between these two groups.

### Histopathological assessment

Paraffin embedding followed by slicing of formalin-fixed bladders and hematoxylin-erythrosine (HE) staining were done using standard procedures at Cellular & Molecular Imaging Core Facility NTNU. HE stained tissues were examined for morphological changes by an uropathologist using a light microscope (Nikon Eclipse 80i).

### Cell viability assay

Cell viability (MTT-assay) was measured as previously described [14]. Data is reported as average ± SD of at least four technical replicas. Data is from one representative experiment out of at least three with similar results.

### *In vitro* cell treatments for microarray, MIB-assay, mass spectrometric metabolic profiling, quantification of extracellular metabolites and comet assay

Um-Uc-3 and T-24 cells were seeded (3-4x10^6^ cells/15 cm plate) and treated with APIM-peptide (8 μM (Um-Uc-3) and 16 μM (T-24)) and cisplatin (10 μM) alone or in combination the next day (three treatment groups and one untreated control per cell line). Extracts from three individual biological replicas (done on different days) were prepared after 24 hours (h) for all conditions of each cell line. The doses were chosen based on the MTT data and the doses given intravenously to rats in the *in vivo* studies (∼1/10 of this dose).

### Microarray- analysis

Samples were prepared as previously described [23]. The microarray experiments have been deposited in the ArrayExpress database (http://www.ebi.ac.uk/arrayexpress) under accession number E-MTAB-5644. Gene expression data was normalized and analyzed using GeneSpring 12.6-GX (Agilent Technologies). DE genes were selected by comparing treated samples to untreated controls, and filtered by flags and fold change ≥1.25. Lists of up- and downregulated genes identified in all three biological replicas of both Um-Uc-3 and T-24 cell lines (n=3+3), and unique for the combination group (not in common with cisplatin group) were extracted. The GeneGo database (MetaCore) was used to annotate these lists of DE genes to gene ontology (GO) pathways.

### MIB-assay

Total cell extracts were prepared as previously described [8]. Kinase enrichment was performed and eluted peptides were analyzed by Orbitrap MS as previously described [41]. The MS proteomics data has been deposited to the ProteomeXchange Consortium (http://proteomecentral.proteomexchange.org) via the PRIDE [42] partner repository with the data set identifier (PXD008724). Label-free quantification values were log-transformed with the base 2 and the transformed control values were subtracted. The resulting values reflecting the change relative to control for each condition were subjected to two-sided Wilcoxon Sign Rank Test [43] as implemented in MATLAB R2015a (Mathworks Inc.). Proteins with p-value <0.25 were considered significantly changed. Three biological replicas were analyzed for each of the treatments. Proteins exhibiting the same trends in both T-24 and Um-Uc-3 cells, and significantly changed in at least one of the cell lines, were selected.

### Quantification of extracellular metabolites

Supernatants were collected, lyophilized and up-concentrated four times in deuterium oxide (Sigma-Aldrich). 1D proton spectra were recorded at 25°C on a Bruker Ascend 400 MHz Avance III HD equipped with a 5 mm Z-gradient SmartProbe (Bruker). The anomeric proton of α-glucose (5.2 ppm), methyl Hβ of lactate (1.3 ppm) and methylene Hγ of glutamine (2.4 ppm) were integrated and quantified by electronic reference to access *in vivo* concentrations (ERETIC2, Topspin 3.5, Bruker). The methylamine H of a creatine (3.0 ppm) external standard (Sigma-Aldrich) was defined as the ERETIC reference. Consumption/production was normalized to average number of live cells (average of live cell density when treatment was initiated and live cell density at time of harvest) within the 24h time interval examined to obtain consumption/production /cell/24h. Four independent cultures of Um-Uc-3 and T-24 cells were analyzed for each condition.

### Targeted mass spectrometric metabolic profiling

Cells were sampled as described in [44], transferred directly to liquid nitrogen and extracted and up-concentrated as described in [45]. Phosphorylated metabolites were prepared for and analyzed by capillary ion chromatography (capIC)-MS/MS as described in [44]. Organic acids were derivatized as described in [46] prior to analysis by liquid chromatography (LC)-MS/MS. Derivatized samples (5 μl) were injected onto a Waters Aquity BEH C_18_ 2.1 x 100 mm column, maintained at 40°C and eluted with mobile phases (A) water added 0,1% formic acid and (B) methanol. The following gradient (v/v%) was applied with a flow rate of 0.25 ml/min: 0-0.5 min; 50% B, 0.5-6 min: 50-99% B, 6-7 min: 99% B, 7-7.1 min: 100-50% B, 8 min: end. Amino acids were derivatized by a protocol adapted from [47], making use of propyl chloroformate and n-propanol, and analyzed by LC-MS/MS. Derivatized samples (1 μl) were injected onto a Phenomenex EZ faast AAA-MS 250 x 0.2 mm column maintained at 25°C and eluted with mobile phases (A) water and (B) methanol, both added 10 mM ammonium formate. The following gradient (v/v %) was applied with a flow rate of 0.25ml/min: 0-1min: 68% B, 1-11min: 68-85% B, 11-11.5min: 85-68% B, 15 min: end. Both LC-MS/MS analyses were performed on a Waters AQUITY UPLC/Xevo TQ-S MS system operated in positive electrospray mode. Absolute quantification from a dilution series of external standards (organic and amino acids, Sigma-Aldrich) was performed in MassLynx V4.1 (Waters). LC-MS/MS analysis was performed for four independent cultures per condition from three biological replicas, capIC-MS/MS analysis was performed for four independent cultures per condition. Metabolome concentrations/abundances were normalized to total ion intensity and tested for significant differences between treatment groups by ANOVA and post hoc Tukey’s range test (p<0.05).

### Alkaline comet assay

Single-cell gel electrophoresis (comet assay) detecting DNA single and double strand breaks, alkali-labile sites, interstrand crosslinks and incomplete excision repair sites, were performed as previously described [48] with minor modifications: Harvested cells were suspended in low melting agarose (1%, 10^5^ cells/mL) and spread on CometAssay^®^ HT slides (Trevigen) (40 μL) in technical duplicates for each condition. Samples were incubated in lysis buffer overnight (4°C) and in alkaline solution (pH>13, 60 min) before gel electrophoresis (0.3A, 30 min). The slides were washed in neutralization buffer (0.4M Tris-HCl), fixed in ethanol and stained with SYBR^®^ Green I (Sigma-Aldrich) before analysis using the Comet Assay IV software (Perceptive Instruments). Cells treated with hydrogen peroxide (100 mM, 20 min, 4°C) were used as a positive control. Fifty comets from each technical duplicate were randomly selected and analyzed for each condition (100 comets) in each biological experiment. Data for all three biological replica is presented (300 comets), and average ± SEM is given. Statistical significance between groups were calculated by student *t*-test (unpaired, two-tailed, ^**^p<0.01, ^***^p<0.0001).

## CONCLUSIONS

**Figure 7:**
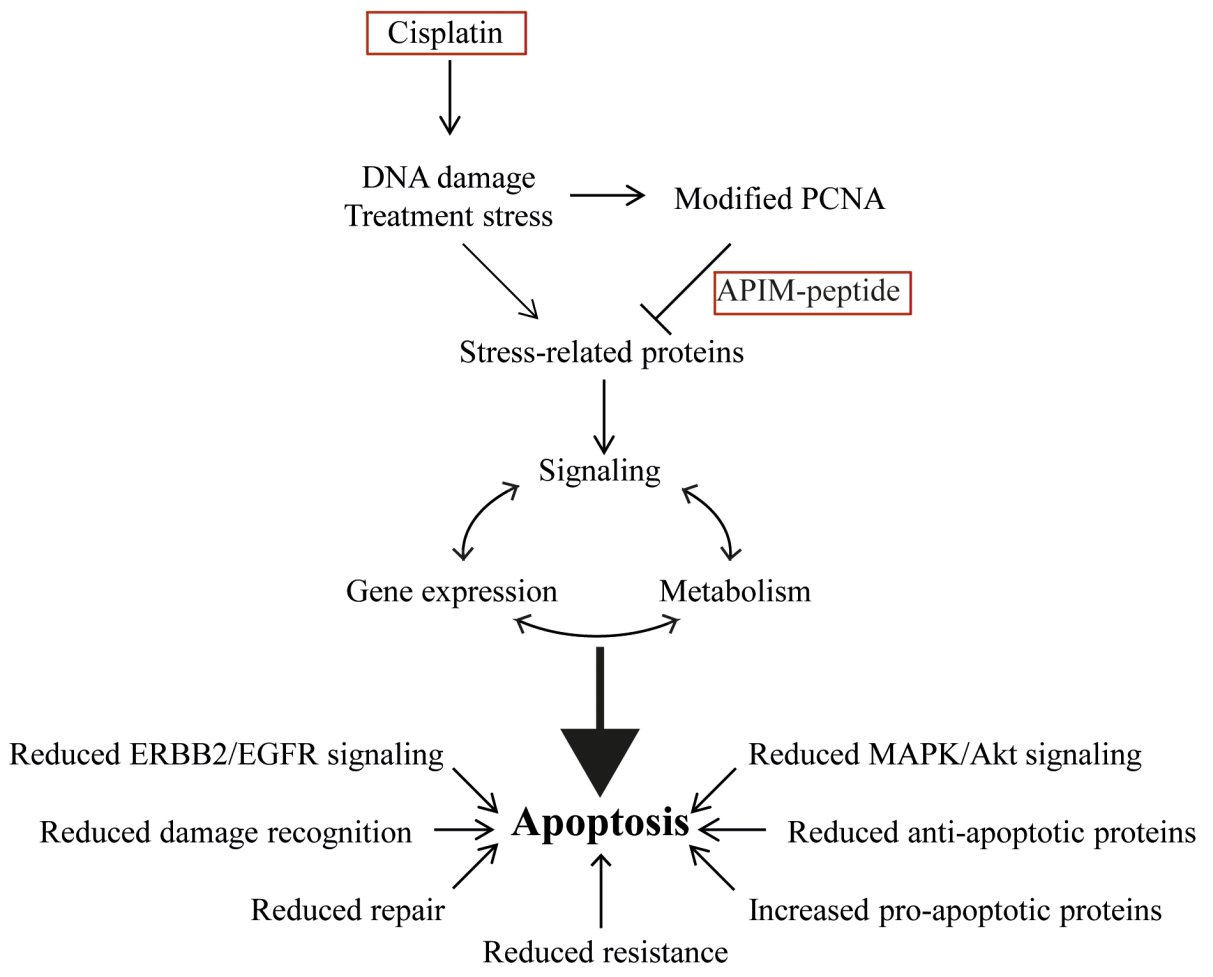
Combination therapy of cisplatin and APIM-peptide produce multiple effects driving the cells towards apoptosis Cisplatin introduces DNA damage and treatment stress that increases the affinity of APIM-containing proteins for PCNA. The APIM-peptide inhibits these interactions, producing alterations in the cells signaling, gene expression profile and metabolism that ultimately pushes the cells towards apoptosis. Reduced EGFR/ERBB2, MAPK and AKT signaling, reduced damage recognition and DNA repair, reduced cisplatin resistance, reduced energy charge and increased expression of pro-apoptotic factors are all contributing to the APIM-peptide-cisplatin combinations mode of action in bladder cancer cells.

In this study we demonstrate an increased anti-cancer efficacy of cisplatin when combined with the PCNA-targeting APIM-peptide, both *in vitro* in human BC cell lines and *in vivo* in the MIBC model. Our results suggest that several key genes and pathways relevant for multiple solid tumors, including MIBC, are affected after treatment with the APIM-peptide-cisplatin combination. In particular, reduced EGFR/ERBB2 signaling, reduced repair of cisplatin-induced DNA damage, re-sensitization of cisplatin-resistant cells and increased apoptosis were features of the combination treatment (summarized in Figure 7). All these changes contribute to the increased anti-cancer efficacy observed for the combination treatment. In conclusion, our results suggest that the APIM-peptide has the potential to improve cisplatin-therapy in the clinical setting and cause an increased anti-cancer response less likely to be circumvented by resistance.

## SUPPLEMENTARY MATERIALS FIGURES AND TABLES







## Abbreviations

APIM: AlkB homologue 2 PCNA interacting motif
BC: Bladder cancer
DE: Differentially expressed
GC: Gemcitabine and cisplatin
MIB-assay: Multiplexed inhibitor bead assays
MIBC: Muscle invasive bladder cancer
MVAC: Methotrexate, vinblastine, adriamycin and cisplatin
NER: Nucleotide excision repair
PCNA: Proliferating cell nuclear antigen
TLS: Translesion synthesis
