# Supplementary material for: “Two hits - one stone”; increased efficacy of cisplatin-based therapies by targeting PCNA’s role in both DNA repair and cellular signaling
Source: Oncotarget. 2018 Aug 21;9(65):32448–65. doi: 10.18632/oncotarget.25963 (PMC6126690; doi:10.18632/oncotarget.25963)
Supplement: Supplementary file 1 [file oncotarget-09-32448-s001.pdf]

“Two hits - one stone”; increased efficacy of cisplatin-based therapies by targeting PCNA’s role in both DNA repair and cellular signaling

SUPPLEMENTARY MATERIALS

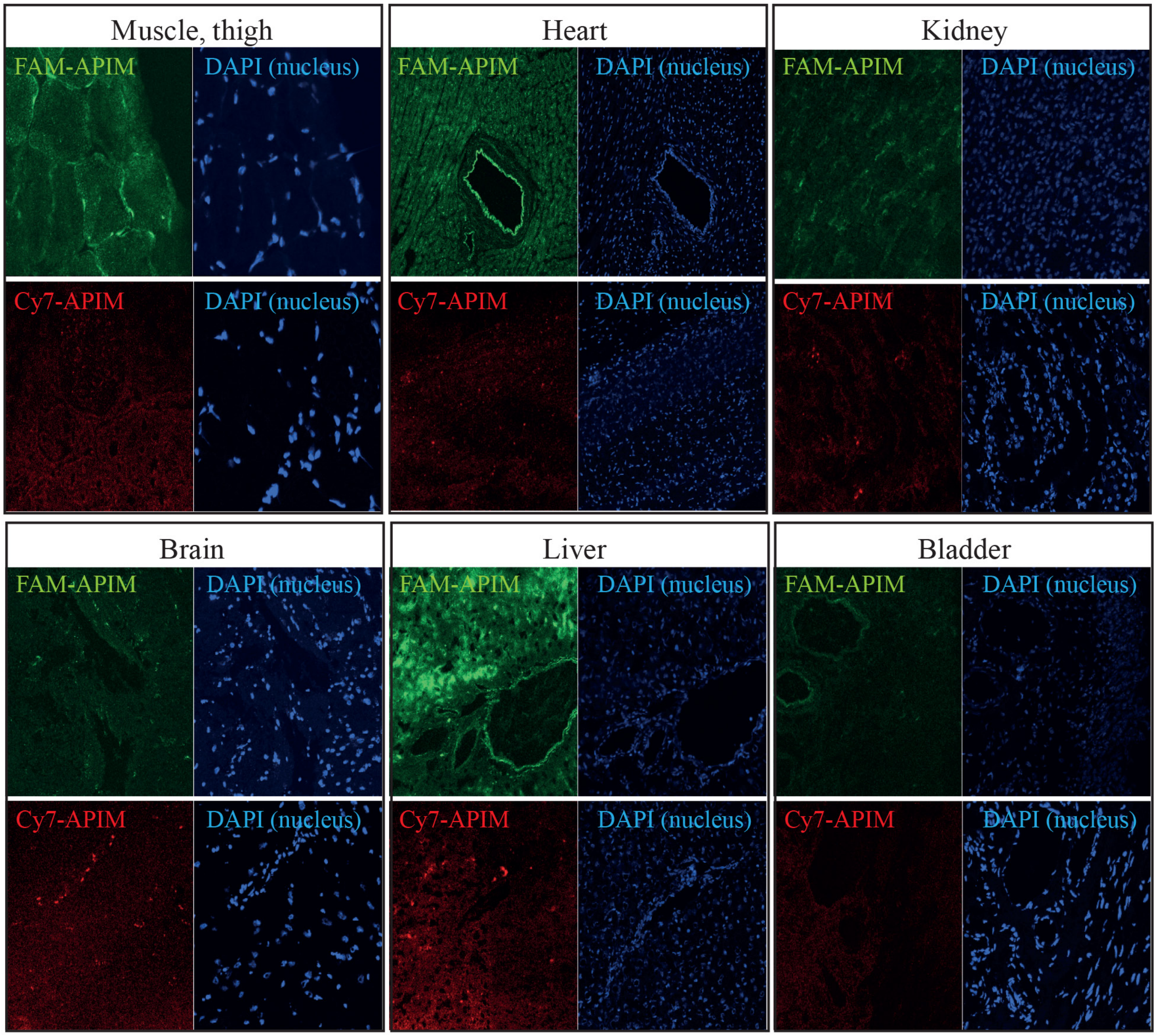

Supplementary Figure 1: APIM-peptide distributes to all organs investigated after intravenous administration. Distribution of FAM-tagged or Cy7-tagged APIM-peptide (9/10 mg net APIM peptide/kg BW in saline) in different organs harvested immediately after i.v. infusion (4 mL/h, 30 min). Frozen sections of thigh muscle, heart, kidney, brain, liver and bladder were DNA-stained with DAPI and evaluated by confocal microscopy using a 20x dry objective. Organs harvested from vehicle treated rats were used as controls.

A

DE genes at 4h

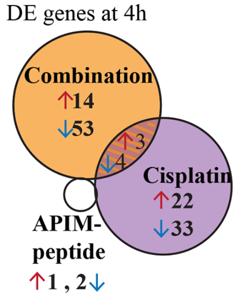

B

Combination DE genes:

Downregulated (↓):

**ADAM9**	**CCNA2**	DIAPH3	FAM83D	KIFC1	PLOD2	TGIF1
ANKRD11	CCNF	DOCK10	FNDC3B	LBR	RAP1GDS1	VEGFC
ASAP2	**CDCA2**	DYRK1A	GNAQ	MEIS2	RPAP3	
ATG4C	**CENPA**	EIF3CL	HEATR3	MGC40489	**SGOL1**	
**AURKA**	CKAP2L	EIF4G2	HYLS1	MRE11A	SLC38A1	
C15orf23	CTCF	EXT1	**INCENP**	NAV2	SNAP23	
C20orf55	DDEF2	FAM48A	**KIF20A**	NEIL3	TANC1	↓

Upregulated (↑):

BTG3	MBLAC2	OKL38	RPL14	RPL9	ZNF296	
FAM162A	NKX2-5	RNU1A3	RPL7	UFM1		↑

C

GeneGo pathway map	Genes in pathway	FDR
**Downregulated:**		
**Cell cycle**		
1. Chromosome condensation in prometaphase	3/21	1E-3
2. The metaphase checkpoint	3/36	3E-3
4. Role of APC in cell cycle regulation	2/32	4E-2
**Signaling**		
3. Transcription factor Tubby signaling pathways	2/17	2E-2
5. G-protein signaling, RhoA regulation pathway	2/34	4E-2
6. Hedgehog and PTH signaling pathways in bone development	2/36	4E-2
11. G-protein signaling, regulation of cAMP levels by ACM	2/45	4E-2
13. Endothelin-1/EDNRA transactivation of EGFR	2/46	4E-2
**Cytoskeleton**		
7. ACM3 and ACM4 in keratinocyte migation	2/38	4E-2
**Development**		
8. Gastrin in differentiation of the gastric mucosa	2/38	4E-2
12. Neurophysiological process, ACM regulation of nerve impulse	2/46	4E-2
**Transport**		
9. ACM in salivary glands	2/42	4E-2
**Apoptosis/Survival**		
10. Anti-apoptotic action of Gastrin	2/43	4E-2
**Upregulated:**		
**Signaling**		
1. Oxytocin receptor signaling	1/40	7E-3
2. Ca2+ dependent NF-AR signaling in cardiac hypertrophy	1/57	7E-3
3. NF-AT signaling in cardiac hypertrophy	1/65	7E-3

D

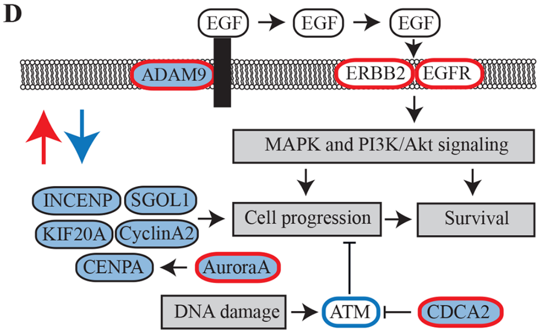

Supplementary Figure 2: APIM-peptide in combination with cisplatin downregulates cell cycle progression genes. Microarray analysis of Um-Uc-3 and T-24 cells treated for 4h with APIM-peptide (8 or 16 µM) and cisplatin (10 µM) alone or in combination relative to untreated controls (n=6). **(A)** Venn diagram illustrating number of DE genes (FC>1.1 compared to untreated control) in each treatment group **(B)** Lists of DE genes identified only in combination treated cells (orange area in A). Duplicate names are removed. **(C)** Significant up- and downregulated GeneGo pathway maps after gene enrichment analysis on the DE genes listed in B. The GeneGo pathway maps are grouped by their main category. **(D)** Schematic overview highlighting the most interesting downregulated DE (blue background, bold in B) genes detected only in the combination group with relevance to MIBC (red edge=overexpressed, blue edge=inactivated). ADAM9 activates and release EGF, a ligand for EGFR and ERBB2, which in turn activates several signaling pathways generally supporting cell cycle progression and survival. Downregulation of CDCA2 results in less inhibition of ATM dependent signaling important during DNA damage response and thereby increases cell cycle arrest. Aurora A phosphorylates CENPA stimulating cell cycle progression. INCENP, SGOL1, KIF20A and cyclin A2 are all important for proper cell cycle progression. **References:** *Knowles et al, Nature reviews cancer, 2015; O'Shea et al, International journal of cancer, 2003; Zhou et al, Clinical cancer research, 2013.*

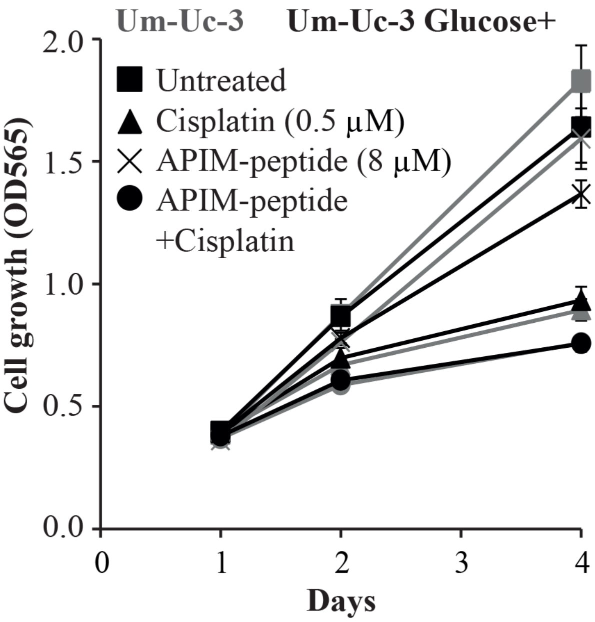

Supplementary Figure 3: Addition of glucose to Um-Uc-3 cells does not affect cell growth. Cell growth of Um-Uc-3 cells with (black) or without (grey) additional glucose in the growth media (10 mg added every 24h) measured over time by the MTT-assay after continuous exposure to the APIM-peptide (8 µM) and cisplatin (0.5 µM) alone or in combination. Data is displayed as average ± SD (4-9 technical replicas) in one representative experiment out of two with the same trends in cell growth.

A

Abbreviation	Metabolite
1,3BPG	1,3-bis-Phosphoglycerate
2HG	2-hydroxyglutarate
2PG	2-Phosphoglycerate
3PG	3-Phosphoglycerate
6PG	6-phosphogluconate
6PGL	D-6-Phosphate-glucono-delta-lactone
AcCoA	Acetyl-CoA
ADP	Adenosine diphosphate
aKG	a-Ketoglutarate
Ala	Alanine
AMP	Adenosine monophosphate
Arg	Arginine
Asn	Asparagine
Asp	Aspartate
ATP	Adenosine triphosphate
CDP	Cytidine diphosphate
Cit	Citrate
CMP	Cytidine monophosphate
CTP	Cytidine triphosphate
Cys	Cystein
dADP	Deoxyadenosine diphosphate
dAMP	Deoxyadenosine monophosphate
dATP	Deoxyadenosine triphosphate
dCTP	Deoxycytidine triphosphate
dGDP	Deoxyguanosine diphosphate
dGTP	Deoxyguanosine triphosphate
DHAP	Dihydroxyacetone phosphate
dIMP	Deoxyinosine monophosphate
dTDP	Thymidine monophosphate
dTMP	Thymidine monohosphate
dTTP	Thymidine triphosphate
dUMP	Deoxyuridine monophosphate
dUDP	Deoxyuridine diphosphate
dUTP	Deoxyuridine diphosphate
E4P	Erythrose 4-phosphate
F1,6BP	Fructose 1,6-bisphosphate
F1P	Fructose 1-phosphate
F6P	Fructose 6-phosphate
Fum	Fumarate
G1P	Glucose 1-phosphate
G6P	Glucose 6-phosphate
GA3P	Glyceraldehyde 3-phosphate
GDP	Guanosine diphosphate
GL3P	Glycerol 3-phosphate
Glc	Glucose
Glc Ex	Glucose extracellular
Gln	Glutamine
Gln Ex	Glutamine extracellular
Glu	Glutamate
Gly	Glycine
GMP	Guanosine monophosphate
GTP	Guanosine triphosphate
Hyp	Hydroxyproline
His	Histidine
Icit	Isocitrate
Ile	Isolecine
IMP	Inosine monophosphate
ITP	Inosine triphosphate
Lac	Lactate
Lac Ex	Lactate extracellular
Leu	Leusine
Lys	Lysine
M1P	Mannose 1-phosphate
M6P	Mannose 6-phosphate
Mal	Malate
Met	Methionine
OAA	Oxaloacetate
Orn	Ornitine
PEP	Phosphoenolpyruvate
Phe	Phenylalanine
Pro	Proline
Pyr	Pyruvate
R5P	Ribose 5-phosphate
RL5P	Ribulose 5-phosphate
S7P	Sedoheptulose 7-phosphate
Ser	Serine
Suc	Succinate
SucCoA	Succinate CoA
Thr	Threonine
Trp	Tryptophan
Tyr	Tyrosine
UDP	Uridine diphosphate
UMP	Uridine triphosphate
UTP	Uridine monophosphate
Val	Valine
X5P	Xylulose-5-phosphate

B

Metabolite class	Metabolite	APIM-peptide / Control						Cisplatin / Control						Combination / Control					
		T-24			Um-Uc-3			T-24			Um-Uc-3			T-24			Um-Uc-3		
		1	2	3	1	2	3	1	2	3	1	2	3	1	2	3	1	2	3
Ex.	Glc Ex			0,0			0,1			0,2			0,5			0,4			0,5
	Gln Ex			0,0			-0,1			0,5			0,8			0,8			0,1
	Lac Ex			0,0			0,1			0,2			0,4			0,2			0,5
Glycolysis, PPP and phosphorylated sugars	PEP			-0,8			0,5			-0,4			-0,1			-0,5			0,3
	GA3P			-0,8			0,6			-0,8			0,0			-1,2			-0,1
	DHAP			-0,3			2,0			1,0			1,0			1,7			2,8
	GL3P			-0,3			0,5			-0,4			0,2			-0,3			0,6
	2PG/3PG			-0,2			0,7			0,3			-0,2			0,5			0,6
	RL5P/R5P/X5P			-0,2			0,9			-0,5			-0,1			-0,2			0,7
	G1P/M1P			-0,8			0,3			-0,5			-0,4			-0,4			0,4
	F1P			-0,9			2,0			1,0			2,0			1,3			3,9
	G6P			-0,6			1,1			-0,3			-0,6			-0,4			0,6
	F6P			-0,6			0,9			-0,2			-0,7			-0,5			0,4
	M6P			-0,5			0,8			0,0			0,0			-0,2			0,5
	6PG			0,0			0,7			-0,5			-1,4			-0,6			-2,1
	F1,6BP			0,0			2,8			0,8			0,8			1,6			3,7
Organic acids	Lac				0,0	-0,1	0,1				0,0	-0,1	0,1				0,1	-0,1	0,0
	Pyr		-0,1	0,2	0,3	0,2	0,2		0,5	0,4	0,6	0,2	-0,2		0,2	0,6	0,2	0,0	0,7
	Fum	-0,1	-0,2	-0,1	-0,7	0,2	0,0	0,0	-0,2	0,2	0,5	-0,1	-0,6	-0,2	-0,4	0,1	0,2	0,2	-0,1
	Suc				0,1	0,2	0,1				0,5	0,3	-0,5				0,2	0,0	0,0
	Mal	0,0	0,0	0,0	0,1	0,1	-0,1	0,2	0,2	0,2	-0,1	0,1	-0,5	0,2	0,2	0,3	-0,5	-0,3	-0,4
	2HG	0,1	0,3	0,4	0,1	0,2	0,0	-0,6	-0,2	-0,7	0,1	-0,1	-0,6	-0,1	0,2	-0,9	0,0	-0,1	-0,1
	OAA				0,2	1,0	-0,5				0,1	-0,6	-0,5				-0,1	-0,5	0,3
	aKG	-0,1	-0,1	-0,2	0,0	-0,3	-0,5	-0,6	-0,4	-0,5	-1,1	-0,6	-1,1	-0,8	-0,4	-1,0	-0,8	0,3	-1,6
	Cit	0,1	0,0	0,1	0,0	0,1	-0,1	0,0	0,1	0,1	0,4	0,6	-0,2	0,2	0,1	0,2	0,0	0,4	0,4
	Icit		0,2	0,1	-0,3	-0,2	-0,4		-0,1	-0,2	0,2	0,5	-0,3		-0,2	-0,7	0,1	0,4	0,2
Amino acids	Gly		-0,1	-0,2	0,0	0,0	-0,4		-0,2	-0,4	0,7	0,4	0,5		-0,3	-0,4	0,5	0,3	0,3
	Ala		-0,1	-0,2	0,1	-0,2	0,3		-0,2	-0,4	-0,3	-0,9	-0,3		-0,3	-0,4	-0,5	-0,8	-0,8
	Ser		-0,1	0,2	-0,3	0,4	-0,1		0,1	0,2	0,0	0,1	-0,2		0,5	0,5	-0,2	0,4	0,2
	Pro		0,0	0,0	-0,1	-0,1	0,0		-0,1	0,1	0,1	0,3	0,3		-0,2	0,0	0,2	0,4	0,3
	Val		0,3	0,1	-0,4	0,0	0,1		0,2	0,2	0,0	-0,1	0,1		0,5	0,5	-0,4	0,0	-0,2
	Thr		0,0	0,1	0,0	0,0	0,2		0,4	0,3	-0,2	-0,1	-0,2		0,3	0,4	-0,2	-0,2	-0,1
	Hyp		-0,2	0,0	0,2	0,0	0,1		-0,4	-0,4	0,0	-0,2	-0,3		-0,6	-0,4	-0,2	-0,5	-0,6
	Leu		0,2	0,1	-0,1	-0,1	0,0		0,2	0,2	-0,1	0,0	0,1		0,4	0,4	0,0	0,1	0,2
	Ile		0,2	0,1	-0,1	-0,2	0,0		0,1	0,1	0,0	-0,1	0,1		0,4	0,3	-0,1	0,0	0,0
	Met		0,0	0,0	0,0	-0,3	0,1		-0,1	0,1	0,1	-0,3	0,0		-0,5	0,2	0,0	-0,3	0,3
	Phe		0,0	0,1	0,0	-0,1	-0,2		0,2	0,2	0,0	0,0	-0,1		0,2	0,2	-0,1	-0,1	0,1
	Asp		-0,1	0,1	0,3	0,1	-0,1		0,3	0,4	-0,1	-0,2	-0,3		0,4	0,3	-0,5	-0,9	-0,6
	Tyr		0,0	0,1	0,0	-0,1	-0,2		0,2	0,2	-0,1	0,0	-0,1		0,1	0,2	-0,1	-0,1	0,0
	Trp			1,3	0,2	0,1	0,0			0,6	-0,2	-0,1	-0,1			1,5	0,1	0,2	0,3
	Orn			0,2	0,0	-0,2	0,2			0,6	0,1	0,1	0,2			0,6	0,1	-0,1	0,6
	Lys		0,4	0,4	-0,1	0,0	0,1		0,8	0,6	-0,1	0,2	0,3		1,3	1,1	0,1	0,2	0,6
Nucleoside phosphates	AMP			-0,6			0,5			0,1			1,4			0,5			1,8
	ADP			0,1			0,2			0,5			0,8			0,6			0,2
	ATP			0,1			-0,4			0,1			0,4			-0,2			-1,2
	GMP			-0,5			-1,3			-0,4			-1,1			-0,3			-2,3
	GDP			-0,1			1,2			0,5			1,2			0,6			0,7
	GTP			0,5			0,6			0,2			0,6			0,3			-0,3
	dTMP			-0,2			-0,5			1,5			1,5			1,5			0,3
	dTDP			-0,2			1,0			1,3			2,2			0,9			1,3
	dTTP			0,1			0,1			1,2			1,1			1,2			0,7
	CMP			-0,3			-0,1			0,1			0,6			-0,1			1,0
	CDP			-0,5			-0,2			-0,2			-0,6			-0,2			0,0
	CTP			-0,8			0,8			-0,5			0,7			-0,3			1,1
	UMP			-0,3			0,0			-0,2			0,0			0,0			0,6
	UDP			-0,4			-0,2			-0,3			-0,6			-0,1			0,0
	UTP			0,4			0,2			-0,7			0,0			-0,6			0,4
	IMP			-0,5			1,1			-0,1			1,4			0,0			0,8
	ITP			0,0			-0,2			-0,1			-0,9			-0,1			-0,6
	dAMP			0,3			-1,2			2,0			1,2			2,1			-0,1
	dADP			-0,2			0,5			1,5			1,9			1,4			1,2
	dATP			0,2			-0,2			1,4			1,0			1,1			0,5
	dGDP			0,4			0,6			1,5			1,9			1,5			1,0
	dGTP			-0,1			-0,2			-0,2			-0,8			-0,1			-0,6
	dCTP			0,4			-0,1			0,8			-0,2			1,1			0,0
	dIMP			-1,0			0,0			0,8			1,9			1,1			1,7
	dUMP			-0,5			-0,8			0,3			0,0			0,4			-0,3
	dUTP			0,9			1,1			0,2			0,8			0,5			-0,5

C

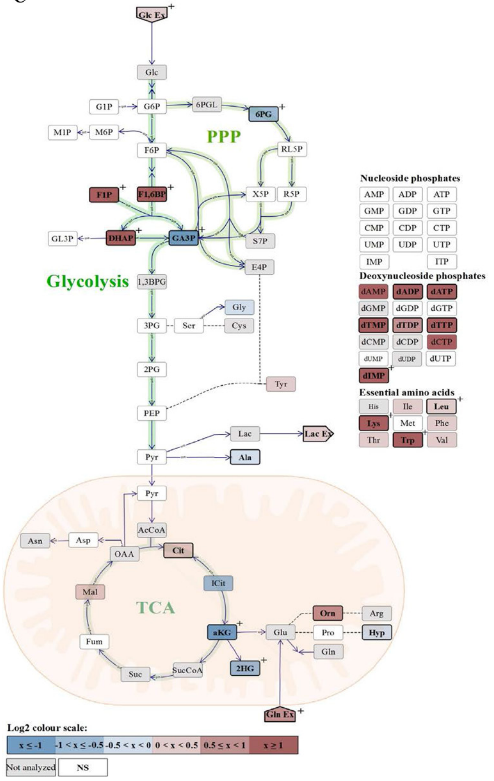

Supplementary Figure 4: Summary of mass spectrometric metabolic profiling. Metabolite levels of T-24 and Um-Uc-3 cells treated with APIM-peptide (16/8 µM), cisplatin (10 µM) or the combination for 24h relative to untreated control. Data is in log2-space. Blue color: decreased metabolite level; red color: increased metabolite level. **(A)** Metabolite abbreviations. **(B)** Table of relative metabolite levels between treatments in both cell lines. The average of four independent cultures is presented for each of three biological replicas. **(C)** Simplified schematic overview of central carbon metabolism covered by targeted metabolic profiling. Levels of significantly changed glycolytic, TCA and PPP intermediates, phosphorylated sugars, amino acids and (deoxy)nucleoside phosphates and consumption/production of glucose, glutamine and lactate in combination treated cell/24h relative to control in T-24 cells (ANOVA and post hoc Tukey's range test, $p<0.05$). The average of four independent cultures from one representative biological replica is presented. Bold metabolites: Reproduced in Um-Uc-3 cells, "+"-denoted metabolites: metabolite level was more (>10%) affected by combination treatment than by cisplatin single-agent treatment.

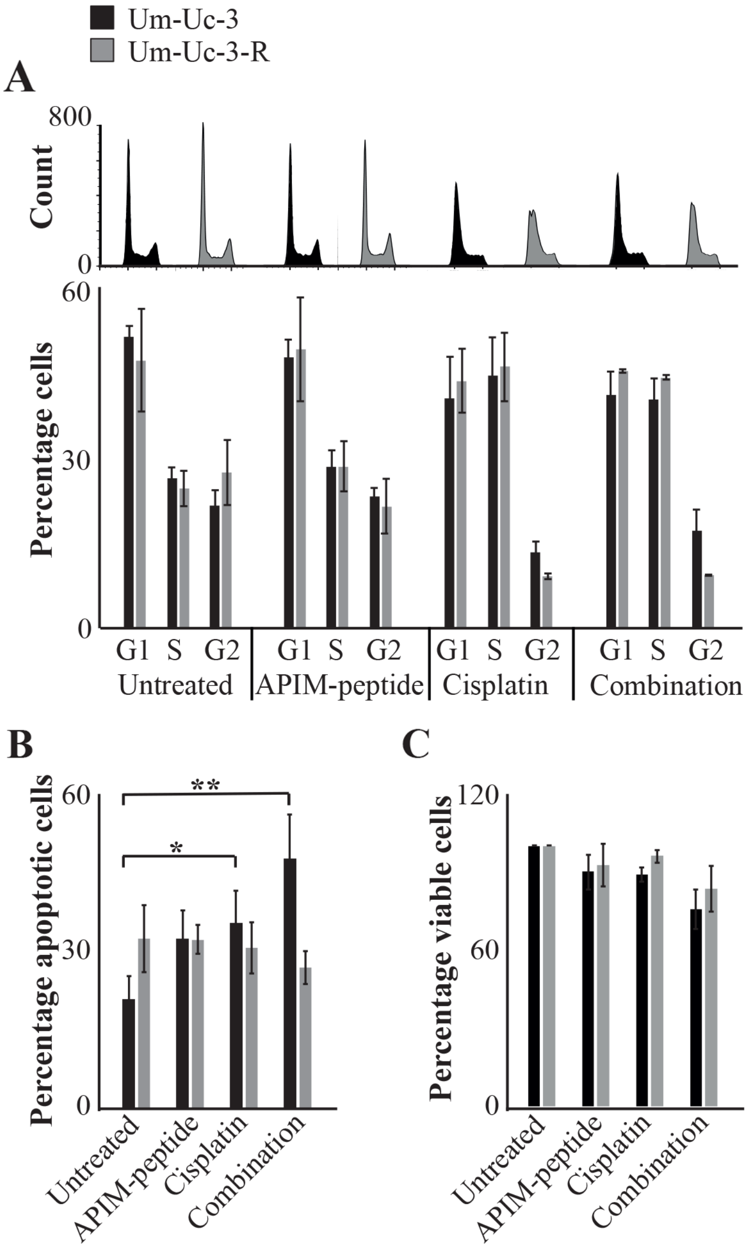

Supplementary Figure 5: Cisplatin induce S-phase arrest in Um-Uc-3 and Um-Uc-3-R cells, while apoptosis only is induced in Um-Uc-3 cells at 24h. Analysis of Um-Uc-3 (black) and Um-Uc-3-R (grey) cells treated for 24h with APIM-peptide (8 µM) and cisplatin (10 µM) alone or in combination. **(A)** Flow cytometry cell cycle analysis performed as previously described (*Søgaard et al, Oncotarget, 2018*). The average percentage cells in each cell cycle phase ± SD (n=3) are presented in the bar graph, with representative cell cycle distribution graphs on top. **(B)** Average percentage apoptotic cells ± SEM (n=6). *p<0.05, **p>0.01 (student-t test, two tailed) measured by flow cytometry as described (*Søgaard et al, Oncotarget, 2018*). **(C)** Average viability ± SEM (n=3) of treated cell relative to untreated cells analyzed with the MTT-assay.

Supplementary Table 1: DE genes detected only in the combination treated group at 24 hours. Lists of DE genes (FC>1.25 compared to untreated control) identified in both Um-Uc-3 and T-24 cells (n=6) and only after APIM-peptide (8/16 µM)-cisplatin (10 µM) combination treatment for 24h. These genes were used for gene enrichment analysis to annotate to significant GO pathway maps. Duplicate gene names are removed. The lists are related to Figure 3 (genes in bold) and Table 2 of the paper. **(A)** Upregulated, **(B)** downregulated.

See Supplementary File 1

Supplementary Table 2: Changed proteins detected by MIB-assay in combination treated group. Lists of significant changed proteins (relative to untreated control, Wilcoxon Sign Rank test, p<0.25) identified by the MIB-assay in both Um-Uc-3 and T-24 cells (n=6, significant for at least one cell line and in the same direction for the other) at 24h after APIM-peptide (8/16 µM) and cisplatin (10 µM) combination treatment. The lists are related to Figure 3 (proteins in bold) and 4 of the paper. **(A)** Upregulated only in combination group, **(B)** downregulated only in combination group. **(C)** Upregulated in cisplatin and in combination group, but more upregulated in combination group (>5% difference), **(D)** downregulated in cisplatin and in combination group, but even more downregulated in combination group (>5% difference).

See Supplementary File 2
