# Supplementary material for: “Two hits - one stone”; increased efficacy of cisplatin-based therapies by targeting PCNA’s role in both DNA repair and cellular signaling
Source: Oncotarget. 2018 Aug 21;9(65):32448–65. doi: 10.18632/oncotarget.25963 (PMC6126690; doi:10.18632/oncotarget.25963)
Supplement: Supplementary file 2 [file oncotarget-09-32448-s002.docx]

**Supplementary Table 1: DE genes detected only in the combination treated group at 24 hours.** Lists of DE genes (FC>1.25 compared to untreated control) identified in both Um-Uc-3 and T-24 cells (n=6) and only after APIM-peptide (8/16 µM)-cisplatin (10 µM) combination treatment for 24h. These genes were used for gene enrichment analysis to annotate to significant GO pathway maps. Duplicate gene names are removed. The lists are related to Figure 3 (genes in bold) and Table 2 of the paper. **(A)** Upregulated, **(B)** downregulated.

**A Upregulated DE genes**

| ABCF2 | CIDECP | GMNN | MRPL18 | RAB15 | SLC6A10P | TMEM170A |
| --- | --- | --- | --- | --- | --- | --- |
| ABHD5 | COPS8 | GOLGA7 | MRPL32 | RAB33B | SLC6A8 | TMEM93 |
| ABL2 | CRY2 | GPATCH4 | MRPS12 | RAE1 | SLFN11 | TMX4 |
| ADRM1 | CXorf40A | GPRC5A | MRPS17 | RANBP1 | SNHG11 | TOB1 |
| AK3 | CYP27B1 | GPRIN1 | MRPS24 | **RASSF1** | SNORA13 | TOB2 |
| AKIRIN1 | DBR1 | GZF1 | MST4 | RBM18 | SNORA26 | TOMM5 |
| ALB | DDX39 | HCST | MTX3 | RBM24 | SNORA28 | TOP3A |
| AMMECR1L | DEDD2 | HIST2H2AA3 | NAP1L5 | RBM34 | SNORA33 | TRIM39 |
| ARL5B | DEM1 | HMGB1 | NARG2 | RBM4 | SNORA57 | TRMT6 |
| ATP6V0B | DGCR6 | HNRNPA2B1 | NAT12 | RCAN1 | SNORA65 | TROVE2 |
| BAG5 | DNAJB4 | IFI30 | NAT13 | RDH5 | SNORD12B | TSC22D1 |
| **BCL2L11/Bim** | DUSP10 | IGFBPL1 | NCDN | RETSAT | SNORD12C | TSEN54 |
| BCL6 | DYNLT1 | IL18BP | NDEL1 | RGS2 | SNORD43 | TSPYL1 |
| BCYRN1 | EAPP | ILF2 | NDUFB9 | RHEBL1 | SNORD46 | TSPYL2 |
| BLOC1S2 | ECE2 | INSM2 | NEURL4 | RIT1 | SNORD49A | TSR1 |
| **BRMS1** | EED | IPO13 | NOC4L | RND1 | SNORD4A | TTC4 |
| BRP44 | EFNB1 | IRX3 | NSUN5 | RNF103 | SNORD52 | TTF1 |
| BRPF1 | EID2 | IRX5 | NSUN5C | RNF114 | SNORD55 | TXNL4B |
| BTG2 | EIF1 | JUNB | NUDT15 | RNU4-1 | SNORD56 | TYRO3 |
| BTN2A1 | EIF2S1 | KBTBD8 | NUP54 | RNU5A | SNORD57 | U2AF1 |
| C12orf31 | EIF4A3 | KCTD6 | OFD1 | RNU6-1 | SNORD69 | UBL7 |
| C12orf57 | EIF4EBP2 | KIAA0773 | OGFRL1 | RNU6-15 | SNRNP27 | UNC119B |
| C14orf138 | EIF5 | KIAA0907 | ORC1L | RPL10L | SNRNP70 | WBP5 |
| C15orf17 | ELOVL4 | KLHL21 | PCBD1 | RPRML | SOD2 | WDR53 |
| C16orf91 | **ENDOG** | LAGE3 | **PCNA** | RSRC2 | STARD13 | ZBTB43 |
| C17orf91 | ENSA | LGMN | PDRG1 | S100PBP | SUPT6H | ZBTB44 |
| C17orf96 | ETS2 | MAD2L1BP | PERP | SAP18 | SYAP1 | ZFAND2A |
| C19orf40 | EXOSC4 | MAD2L2 | PGRMC1 | SAR1B | TAF9 | ZNF124 |
| C1orf55 | FABP5L2 | MAFF | PHACTR4 | SCAND3 | TAP1 | ZNF200 |
| C1orf63 | FAM101B | MAFG | PHLDA2 | SCNM1 | TBCC | ZNF211 |
| C3orf58 | FAM111B | MARCKSL1 | PKMYT1 | SDCCAG3 | TBP | ZNF23 |
| C5orf22 | FAM18B | MASTL | PLEKHO2 | SELK | TBRG4 | ZNF286A |
| C6orf66 | FAM86A | MBD1 | PLIN2 | SERINC1 | TBX2 | ZNF286C |
| **CASP3** | FAM89A | MCOLN1 | **PMAIP1/NOXA** | SFTA1P | TCP1 | ZNF503 |
| CCDC137 | FBXO45 | MEA1 | PNRC2 | SGK | TDG | ZNF567 |
| CCL20 | FBXO5 | MED31 | POLR2A | SGK1 | TDP1 | ZNF598 |
| CCNE1 | FEN1 | METTL13 | POLR2C | SIK1 | TFB2M | ZNF689 |
| CCT6A | FSTL3 | METTL6 | POLR2D | SLBP | THAP1 | ZNF695 |
| CDC2 | GABARAPL1 | MICB | POLR3D | SLC10A3 | THAP10 | ZNF700 |
| CDH24 | GADD45B | MIR1275 | PPIF | SLC25A15 | TICAM1 | ZNF761 |
| CEBPA | GADD45GIP1 | MIR1974 | PPPDE2 | SLC25A28 | TJAP1 | ZNF768 |
| CHCHD7 | GATA2 | MIR1978 | PRIC285 | SLC35A4 | TMEM106C | ZNFX1 |
| CHERP | GDI1 | MIR302C | PRPF4 | SLC39A1 | TMEM132A | ZWINT |
| CHMP5 | GGH | MLF1 | PSME2 | SLC39A6 | TMEM167A |  |

**B Downregulated DE genes**

| ABCC4 | DENND2A | GRK5 | LSM14A | PCCA | RPRD2 | TMEM116 |
| --- | --- | --- | --- | --- | --- | --- |
| ABR | DENND5A | GSK3B | LTBP2 | PCNT | **RPS6KA2/RSK** | TMEM118 |
| ACAP2 | DENND5B | GTF2F2 | M6PRBP1 | PCNX | RRAS2 | TMEM131 |
| ACSL3 | DET1 | GTF2IP1 | MACF1 | PDCD6IP | RRBP1 | TMEM135 |
| ACTA2 | DFFB | GTF3C2 | MALL | PDE6D | RSRC1 | TMEM165 |
| ACTN1 | DFNA5 | GTPBP6 | MAN2B2 | PDGFC | RSU1 | TMEM44 |
| ACVR1 | DHRS1 | H1F0 | MANBA | PDGFRL | RTN4IP1 | TMEM49 |
| ADARB1 | DHRS3 | HAGH | **MAP2K4** | PDIA5 | RXRA | TMEM50B |
| AGRN | DHX15 | HDDC3 | **MAP2K5/**  **MEK5** | PDK3 | RYK | TMLHE |
| AHNAK | DIAPH2 | HDHD1A | MAP3K4 | PDS5A | SAMM50 | TNFAIP8L1 |
| AKAP7 | DIAPH3 | HEATR2 | MAP3K5 | PDS5B | SAPS3 | TNFRSF1A |
| AKTIP | DIP2B | HEATR5B | **MAP4K4** | PDSS2 | SASH1 | TNFRSF21 |
| ALAD | DLGAP5 | HECTD1 | MAP4K5 | PDXK | SBF2 | TNRC15 |
| ALDH6A1 | DNAJC10 | HERC1 | MAPKAP1 | PECR | SCAMP1 | TNRC6B |
| ALG9 | DNAJC13 | **HERC2** | MARCH4 | PEMT | SCARB1 | TNS3 |
| ANAPC10 | DOCK1 | HIBADH | MAT2A | PEPD | SCARB2 | TOX2 |
| ANKHD1 | DOCK10 | HIBCH | MBTPS1 | PET112L | SCD5 | TPM1 |
| ANKIB1 | DOCK7 | HIPK2 | MCPH1 | PEX11B | SCMH1 | TPST2 |
| ANO6 | DOPEY2 | **HK1** | MCRS1 | PEX14 | SDC1 | TRAK1 |
| ANXA11 | DPYD | **HK2** | MCTP1 | **PFKFB4** | SDCCAG10 | TRAK2 |
| APPL2 | DPYSL2 | HMBS | MED22 | **PFKP** | SEC23A | TRAM2 |
| ARF3 | DTD1 | HMGA2 | MED27 | PFTK1 | SEMA3A | TRIM33 |
| ARFGEF1 | DYM | HNRNPA3 | MEIS2 | PGM1 | SEMA4B | TRIM44 |
| ARHGAP10 | DYNC1I2 | HNRNPD | MEMO1 | PHACTR2 | SEMA4D | TRIOBP |
| ARHGAP12 | DYNC2H1 | HNRNPH3 | MERTK | PHCA | SEPT10 | TRPT1 |
| ARHGAP17 | EEF2K | HNRNPK | METTL9 | PHF2 | SEPT2 | TRRAP |
| ARHGAP19 | EFHA1 | HNRNPR | MGC18216 | PHF21A | SEPT6 | TSPAN4 |
| ARHGAP21 | EFHD2 | HNRPA1P4 | MGMT | PHF3 | SEPT9 | TSPAN5 |
| ARHGAP23 | **EGFR** | HNRPA2B1 | MID1 | PHKB | SERGEF | TSPAN9 |
| ARHGEF10 | EHBP1 | HNRPH3 | MIF4GD | PIAS1 | **SETD2/HYPB** | TSSC1 |
| ARHGEF7 | EIF4G3 | HNRPK | MIPEP | PIAS2 | SH3D19 | TTC15 |
| ASAP1 | ELMO2 | HNRPR | MIPOL1 | PIGK | SH3KBP1 | TTC23 |
| ASB8 | ELOVL6 | HOXB8 | MKLN1 | PIGN | SHB | TTC27 |
| ASCC3 | ELP4 | HPCAL1 | MLLT10 | PIGU | SIK3 | TTLL5 |
| ASPSCR1 | EML4 | HPS3 | MLPH | **PIK3CB** | SIL1 | TTYH3 |
| ASXL1 | ENAH | HRASLS3 | MPRIP | PIK4CA | SKAP2 | TULP4 |
| ATG4C | ENG | HSD17B12 | MRPL1 | PIP4K2A | SLC20A2 | TYW1 |
| **ATM** | ENOX2 | HSD17B4 | MRPL20 | PIR | SLC23A2 | TYW1B |
| ATP2C1 | EPB41L2 | HSPA5 | MRPS27 | PJA2 | SLC25A13 | U1SNRNPBP |
| ATXN2 | EPS15 | IARS2 | MRPS28 | PKP4 | SLC25A26 | UBAC2 |
| AUH | **ERBB2** | ID1 | **MSH3** | PLEKHA2 | SLC25A42 | UBE2E2 |
| B4GALT5 | ERC1 | IDE | MSRA | PLOD1 | SLC27A1 | UBE2E3 |
| BCAR3 | ERGIC1 | IDH1 | MSRB3 | PLOD2 | **SLC2A1/GLUT1** | UBE2F |
| BCAS4 | ERI3 | IFI44 | MT1A | PLS3 | SLC30A9 | UBE2G1 |
| BCAT1 | ERICH1 | IFT52 | MT1G | PLXNA1 | SLC35D2 | UBE2L3 |
| **BCL2** | ERLIN2 | IGF2R | MT2A | PNPLA6 | SLC37A4 | UBE4B |
| **BCL2L1/**  **BCLXL** | ETV6 | IL13RA1 | MTA3 | **PODXL** | SLC39A10 | UBR3 |
| BMPR1A | EVI1 | INO80 | MTE | POLA1 | SLC39A11 | UBTD1 |
| C11orf70 | EVI5 | INPP4B | MTHFD1L | POLR3B | SLC41A3 | UGCGL2 |
| C12orf51 | EVI5L | INPP5A | MTIF3 | POTEF | SLC44A1 | UNC84B |
| C14orf102 | EVL | INSIG2 | MTMR10 | PPA2 | SLMAP | UQCC |
| C14orf106 | EXD2 | INTS2 | MTMR2 | PPAP2B | SMAD3 | USO1 |
| C14orf131 | EXOC4 | INTS4 | MTMR4 | PPARG | SMARCA1 | USP13 |
| C14orf159 | EXOC6 | INTS9 | MTSS1 | PPFIA1 | SMARCA2 | USP24 |
| C14orf179 | EXT1 | INVS | **MVP** | PPFIBP1 | SMARCAL1 | USP34 |
| C14orf43 | EXT2 | IPO11 | MYH10 | PPHLN1 | SMARCC1 | USP48 |
| C16orf35 | FAF1 | IPO8 | MYH9 | PPP1CB | SMS | USP6NL |
| C17orf61 | FAM117B | IQCK | MYO10 | PPP2R5C | SMYD2 | USP9X |
| C1GALT1C1 | FAM125B | ITCH | MYO5A | PPP2R5E | SMYD3 | UVRAG |
| C1orf85 | FAM127C | ITFG1 | MYO9A | PPP3CB | SND1 | VAC14 |
| C20orf194 | FAM129B | **ITGA3** | MYO9B | PRAGMIN | SNUPN | VAV2 |
| C2orf30 | FAM171A1 | **ITGB1** | MYOF | PRDM10 | SNX25 | VCL |
| C9orf5 | FAM20C | **ITGB5** | NAP1L1 | PREP | SORT1 | **VEGFC** |
| CAMK2G | FAM38A | ITPR3 | NARG1 | PRKAG2 | SP1 | VEZF1 |
| CAPRIN1 | FAR2 | ITSN1 | NAV1 | **PRKCA** | SPA17 | VPS28 |
| CARM1 | FARP1 | **JAK1** | NAV2 | PRKCZ | SPAST | VPS41 |
| CAV2 | FARS2 | JARID2 | NCK1 | PRMT3 | SPATS2L | VPS45 |
| CCDC109B | FAT1 | KAT2B | NCK2 | PRNPIP | SPG11 | VPS8 |
| CCDC6 | FBXL2 | KDM5B | NCKAP1 | PRR16 | SPIRE1 | WASF3 |
| CCNG1 | FBXL20 | KHDRBS1 | NCOA1 | PRRC1 | SPTBN1 | WBSCR27 |
| CD46 | FBXO11 | KIAA0146 | NCOA6 | PRSS23 | SRBD1 | WDR1 |
| CD47 | FBXW11 | KIAA0182 | NCOR2 | PSMG2 | SRI | WDR19 |
| CDC14B | FCHSD2 | KIAA0196 | NDE1 | PSRC1 | SSH2 | WDR22 |
| CDC2L6 | FER | KIAA0240 | NDRG3 | **PTK2/FAK1** | SSU72 | WDR25 |
| CDCP1 | FER1L3 | KIAA0261 | NDUFAF2 | PTPN4 | ST3GAL3 | WDR4 |
| CDK5RAP2 | FHOD3 | KIAA0319L | NDUFS4 | PTPRA | ST7 | WDR51A |
| CDK6 | FIBP | KIAA0391 | NEDD4L | PTPRF | STAG1 | WDR59 |
| CENPF | FIG4 | KIAA0556 | NEK1 | PTPRG | **STAT6** | WDR60 |
| CENTB2 | FKBP9L | KIAA0564 | NEK6 | PTPRK | STAU2 | WDR7 |
| CEP192 | FKSG30 | KIAA0586 | NFIA | PUM1 | STIM1 | WDR70 |
| CEP63 | FLJ10986 | KIAA1671 | NFIB | PUM2 | STK24 | WNT5B |
| CERK | FLNB | KIAA1688 | NFIC | RAB10 | STK3 | WSCD1 |
| CHCHD3 | FLOT2 | KIAA1797 | NFIX | RAB11A | STK39 | XPO1 |
| CHD9 | FNBP1 | KIF11 | NICN1 | RAB11FIP1 | STMN3 | XPO4 |
| CHM | FNDC3B | KIF1B | NIN | RAB11FIP3 | STS-1 | XPR1 |
| CHN1 | FNIP1 | KIF20B | NIPSNAP1 | RAB40B | STT3B | XRCC5 |
| CLCN6 | FNTB | KIFAP3 | NME7 | RAB40C | STX8 | XYLT2 |
| CLIC4 | FOXJ3 | KLF12 | NOTCH2 | RAB7A | SUCLG2 | **YAP1** |
| CLIP4 | FOXK1 | LACTB | NPEPPS | RABEP1 | SUMF1 | **YWHAE** |
| CLUAP1 | **FRAP1/mTOR** | LAMA5 | NSF | RABGAP1 | SUOX | **YWHAZ** |
| CNN2 | FRMD4A | LAMC1 | NSMCE1 | RAI1 | SUPT3H | ZAK |
| CNOT1 | FRYL | LAMP2 | NSMCE2 | RAI14 | SVIL | ZBTB4 |
| CNOT2 | FTO | LANCL1 | NT5DC1 | RALA | SYNJ2BP | ZC3H3 |
| CNOT6 | FUT8 | LARP7 | NUDT7 | RALGAPA1 | SYT15 | ZCCHC11 |
| CNPY4 | FYCO1 | LARS2 | NUMB | RAPGEF1 | TACC2 | ZCCHC14 |
| COG6 | FZD2 | LASS6 | NUP133 | RAPGEF6 | TAF1B | ZFHX3 |
| COPZ1 | GAB2 | LCLAT1 | NUP37 | RARA | TAF4 | ZFP161 |
| COQ2 | GABBR2 | LCMT1 | NXN | RASA1 | TANC1 | ZFP91 |
| COQ5 | GALK2 | LEPREL1 | ODZ3 | **RB1** | TANC2 | ZFYVE26 |
| COX10 | GALNT10 | LHFP | OPA1 | RBBP4 | TASP1 | ZHX3 |
| CPD | GAS6 | LHFPL2 | OSBPL10 | RBL2 | TBC1D16 | ZMAT5 |
| CREB3L2 | GBE1 | LIMCH1 | OSBPL1A | RBMX | TBC1D19 | ZMIZ1 |
| CRIM1 | GLB1 | LIPA | OSBPL3 | REEP5 | TBC1D22A | ZMYM4 |
| CRTC3 | GLCE | LMBR1 | OSBPL5 | RER1 | TBC1D9B | ZMYND8 |
| CS | GLG1 | LMBRD1 | OSBPL9 | **REV1** | TBCD | ZNF277 |
| CSNK1G1 | GLTSCR1 | LMNA | OSTF1 | RFTN1 | TBCE | ZNF323 |
| CTBP1 | GMDS | LMNB1 | P4HA2 | RFWD2 | TBL1X | ZNF362 |
| CTTN | GNA12 | LONP2 | PACSIN2 | RFX7 | TBL1XR1 | ZNF395 |
| CUEDC1 | GNAQ | LPAR1 | PAFAH1B1 | RGS12 | TCF12 | ZNF407 |
| CUL2 | GNB1 | LPCAT3 | PAM | RHBDF2 | TCF25 | ZNF609 |
| CUL4B | GNB1L | LPP | PAN3 | RHOT1 | TEX2 | ZNF618 |
| CWF19L2 | GNG12 | LRBA | PARD6A | RIN2 | TGFBR2 | ZNF650 |
| DAZAP1 | GNS | LRP5 | PARN | RNASET2 | TGFBR3 | ZYG11B |
| DBNDD1 | GOLGA3 | LRP8 | PARVA | RNF145 | THADA | ZZEF1 |
| DBNL | GPHN | LRRC1 | PARVB | RNF216 | TIAF1 | ZZZ3 |
| DCAF7 | GPR177 | LRRC20 | PBX3 | RNFT2 | TKT |  |
| DDIT4 | GPR56 | LRRC28 | PC | RNGTT | TM9SF4 |  |
| DDX10 | GRB10 | LRSAM1 | PCBP2 | ROD1 | TMED10 |  |
