# Supplementary material for: “Two hits - one stone”; increased efficacy of cisplatin-based therapies by targeting PCNA’s role in both DNA repair and cellular signaling
Source: Oncotarget. 2018 Aug 21;9(65):32448–65. doi: 10.18632/oncotarget.25963 (PMC6126690; doi:10.18632/oncotarget.25963)
Supplement: Supplementary file 3 [file oncotarget-09-32448-s003.docx]

**Supplementary Table 2: Changed proteins detected by MIB-assay in combination treated group.** Lists of significant changed proteins (relative to untreated control, Wilcoxon Sign Rank test, p<0.25) identified by the MIB-assay in both Um-Uc-3 and T-24 cells (n=6, significant for at least one cell line and in the same direction for the other) at 24h after APIM-peptide (8/16 µM) and cisplatin (10 µM) combination treatment. The lists are related to Figure 3 (proteins in bold) and 4 of the paper. **(A)** Upregulated only in combination group, **(B)** downregulated only in combination group. **(C)** Upregulated in cisplatin and in combination group, but more upregulated in combination group (>5% difference), **(D)** downregulated in cisplatin and in combination group, but even more downregulated in combination group (>5% difference).

**A Upregulated proteins only by APIM-peptide-cisplatin treatment**

| ADSL | DGCR14 | GLYR1 | MAGOHB | PPP1R18 | SMARCB1 | XPA |
| --- | --- | --- | --- | --- | --- | --- |
| ALG2 | DHRS4 | GMPPA | MED31 | PRCP | SMC3 | TRPV1 |
| ASNS | DIDO1 | GOLGB1 | MPHOSPH6 | PRPF4 | SNRNP40 | TXNDC12 |
| ATF1 | DNAJC9 | GORASP2 | MRPL15 | RAB21 | SNW1 | TXNDC5 |
| ATP5B | EXOSC8 | HMGB3 | MRPL39 | RAD50 | SPAG1 | UBTF |
| ATP5O | FAM134A | HSPA5 | MRPL40 | RBBP4 | SRSF10 | WDR18 |
| BAG5 | FAM169A | HSPA6/7 | MTHFD2 | RBM14 | SSB | XPA |
| BCS1L | FAM50 | HSPA9 | MTPAP | RCC1 | STRAP | ZNF638 |
| BNIP1 | FARSA | HYOU1 | NCAPH2 | RNF169 | SYNCRIP |  |
| CLPTM1L | FLYWCH2 | IRF2BP2 | NENF | RPN2 | TFAM |  |
| CLSPN | FXR1 | KDM3A | OGDH | RPS14 | TIAL1 |  |
| COLGALT1 | GCC1 | KIAA1217 | PDRG1 | RPSA | TOMM34 |  |
| CREB1 | GLS | LRCH4 | PICALM | SDF2L1 | TPT1 |  |
| DDX5 | GLUD1/2 | LRRC47 | POLR2L | SF1 | TRABD |  |

**B Downregulated proteins only by APIM-peptide-cisplatin treatment**

| ABR | CHMP4B | EIF3E | IQGAP1 | PSMB4 | SMEK1/2 | TNPO1 |
| --- | --- | --- | --- | --- | --- | --- |
| ANP32E | COPS2 | EIF3L | KIDINS220 | RBM19 | SSSCA1 | VAC14 |
| AP3M2 | COPS5 | EXOC7 | MAVS | RNF138 | **STAT3** | VPS16 |
| ARFGAP1 | CTDP1 | FAM120A | MLLT4 | RPLP1 | **STAT6** | WDR81 |
| ARHGEF40 | CUL3 | GSDMD | NAP1L4 | RPS13 | TCP11L1 | WRN |
| ATP2B4 | DDX58 | GSPT1 | NAPG | RSF1 | TFCP2 | XPOT |
| C14orf166 | DNMBP | HAT1 | PDIA4 | SLK | TFPT | YBX1 |
| CDK5 | EIF3D | HSP90AB1 | PPIH | SMARCA2 | TM9SF2 | ZFYVE19 |

**C Proteins more upregulated in APIM-peptide-cisplatin than cisplatin treatment**

| ABHD10 | CDCA3 | DLAT | HNRNPA3 | MAT2A | PAPOLA | SF3A2 |
| --- | --- | --- | --- | --- | --- | --- |
| ACOT9 | CDK9 | DNAJB1 | HNRNPAB | MAT2B | PCBP1 | SLC4A1AP |
| AIFM1 | CHAF1B | DNAJC8 | IDH3B | MAX | PES1 | SUPT6H |
| AIMP1 | CHAMP1 | DNTTIP2 | IFI35 | MBLAC2 | PNPT1 | SUPV3L1 |
| AK3 | CNPY2 | DTX3L | IK | MED17 | PPAT | TCERG1 |
| ALDH1B1 | CORO1C | ECHS1 | KDM2A | MKI67 | RBM4 | TONSL |
| ANXA1 | CPSF1 | EXOSC4 | KIF23 | MMS19 | RBM42 | TRMT6 |
| ANXA4 | CRNKL1 | FBL | KIF2C | MNT | RETSAT | TXNRD1 |
| ARF5 | CUL1 | FDXR | LAMTOR1 | MRPL3 | RPL3 | TYMS |
| ATXN2L | DDX18 | FKBP4 | LRRC41 | MRPL45 | RPL9 | VIM |
| BAZ1B | DDX19 | GMPS | LSM4 | MRPS24 | RPS3A | WDR3 |
| BCAS2 | DDX47 | GNL3 | LUC7L | MSN | RPS4X | WDR36 |
| BUB3 | DECR1 | GNPDA1 | LUC7L3 | NCL | RPS7 | YBX3 |
| C17orf80 | DHX38 | HADHB | MAFF | NO66 | RTCB |  |
| C1QBP | DHX8 | HCCS | MAGOH | NOL6 | SART1 |  |
| CCNT1 | DIS3 | HIP1R | MARCKSL1 | NUP62 | SCP2 |  |

**D Proteins more downregulated in APIM-peptide-cisplatin than cisplatin treatment**

| ABCF3 | CAMSAP2 | DYNC1H1 | MAP2K3 | PPP3CA | SRPK2 | YTHDF2 |
| --- | --- | --- | --- | --- | --- | --- |
| ACY1 | CAP2 | **EGFR** | **MAP4K4** | PPP6R3 | ST13 | ZC3H7A |
| ACACA | CAPN2 | EIF2D | **MAPK1/ERK2** | PRKCDBP | STAG2 | ZFYVE16 |
| ACTN4 | CCDC53 | EIF5B | MAPK14 | PRKDC | STK4 | ZZEF1 |
| ADD1 | CCDC6 | ELF1 | **MAPK3/ERK1** | **PTK2/FAK1** | STUB1 |  |
| AKAP12 | CDC42BPB | EML4 | MGA | PTPN12 | SYAP1 |  |
| AKAP13 | CDK17 | FAM134C | MICAL2 | RABGAP1 | TBC1D9B |  |
| AMPD2 | CEP170B | FAM175B | MICU2 | RNF213 | TMEM263 |  |
| ANAPC7 | CKAP5 | FAM21C | MYO9B | **RPS6KA3** | TRIO |  |
| ANKHD1 | CLPX | FASN | NAGK | RRBP1 | TTC37 |  |
| ANKRD50 | CNOT1 | FER | NAV1 | SEC16A | TUBGCP2 |  |
| AP1M1 | CNOT2 | FRYL | NCAPG | SEC23IP | UBE4B |  |
| AP3B1 | COL4A3BP | GBF1 | NEDD4L | SEC24D | USO1 |  |
| AP3S1 | COPA | GOLGA3 | NEK9 | SIX5 | USP25 |  |
| ARCN1 | CSE1L | GTF2I | OSBPL10 | SNX1 | USP47 |  |
| ARFGEF1 | CSK | HECTD1 | **PAK4** | SNX15 | USP5 |  |
| ARFGEF2 | CTNNA1 | IPO8 | PDCD10 | SNX21 | USP9X |  |
| ARHGAP35 | CTNND1 | IRF9 | PDXDC1 | SNX27 | UVRAG |  |
| ARHGEF10 | DCTN2 | **JAK1** | PELO | SNX5 | VMA21 |  |
| ARL4C | DFNA5 | KIAA1468 | PLIN3 | SNX8 | VPS53 |  |
| BAIAP2 | DIAPH3 | KIF15 | POLR2B | SOX9 | WDR70 |  |
| BIRC6 | DNAAF5 | KPNA6 | POLR3D | SPATA5 | WNK1 |  |
| CAMK2D | DST | MAN2A1 | PPP2R5B | SRGAP1 | **YAP1** |  |
